# Vertical stratification of insect abundance and species richness in an Amazonian tropical forest

**DOI:** 10.1038/s41598-022-05677-y

**Published:** 2022-02-02

**Authors:** Dalton de Souza Amorim, Brian V. Brown, Danilo Boscolo, Rosaly Ale-Rocha, Deivys Moises Alvarez-Garcia, Maria Isabel P. A. Balbi, Alan de Marco Barbosa, Renato Soares Capellari, Claudio José Barros de Carvalho, Marcia Souto Couri, Rodrigo de Vilhena Perez Dios, Diego Aguilar Fachin, Gustavo B. Ferro, Heloísa Fernandes Flores, Livia Maria Frare, Filipe Macedo Gudin, Martin Hauser, Carlos José Einicker Lamas, Kate G. Lindsay, Marco Antonio Tonus Marinho, Dayse Willkenia Almeida Marques, Stephen A. Marshall, Cátia Mello-Patiu, Marco Antônio Menezes, Mírian Nunes Morales, Silvio S. Nihei, Sarah Siqueira Oliveira, Gabriela Pirani, Guilherme Cunha Ribeiro, Paula Raille Riccardi, Marcelo Domingos de Santis, Daubian Santos, Josenilson Rodrigues dos Santos, Vera Cristina Silva, Eric Matthew Wood, José Albertino Rafael

**Affiliations:** 1 Entomology Grad Program, Department of Biology, FFCLRP, University of Sao Paulo, São Paulo, SP Brazil; 2 Urban Nature Research Center and Entomology Section, Natural History Museum of Los Angeles County Los Angeles, Los Angeles, CA USA; 3 Coordenação de Biodiversidade, Instituto Nacional de Pesquisas da Amazônia, Manaus, AM Brazil; 4 University of Sucre, Sincelejo, Colombia; 5 Instituto Federal do Triângulo Mineiro-Campus Uberaba, Uberaba, MG Brazil; 6 Departamento de Zoologia, Universidade Federal do Paraná, Curitiba, PR Brazil; 7 Museu Nacional, Universidade Federal do Rio de Janeiro, Rio de Janeiro, RJ Brazil; 8 Departamento de Zoologia, Instituto de Biociências, Universidade de São Paulo, Sao Paulo, SP Brazil; 9 Universidade Federal da Paraíba, João Pessoa, PB Brazil; 10 Plant Pest Diagnostics Branch, California Department of Food and Agriculture, Sacramento, MG Brazil; 11 Museu de Zoologia, University of Sao Paulo, São Paulo, SP Brazil; 12 School of Environmental Sciences, University of Guelph, Guelph, Canada; 13 Departamento de Ecologia, Zoologia e Genética, Instituto de Biologia, Universidade Federal de Pelotas, Capão do Leão, RS Brazil; 14 Programa de Pós-Graduação em Entomologia, Universidade Federal de Lavras, Lavras, MG Brazil; 15 Department of Ecology, Institute of Biological Sciences, University of Goiás, Goiás, GO Brazil; 16 Centro de Ciências Naturais e Humanas, Universidade Federal do ABC, Santo André, SP Brazil; 17 California State University Los Angeles, Los Angeles, CA USA; 18 Ornithology Section, Natural History Museum of Los Angeles County Los Angeles, Los Angeles, CA USA

**Keywords:** Entomology, Evolutionary ecology, Tropical ecology, Evolution, Zoology

## Abstract

Tropical forests are among the most biodiverse biomes on the planet. Nevertheless, quantifying the abundance and species richness within megadiverse groups is a significant challenge. We designed a study to address this challenge by documenting the variability of the insect fauna across a vertical canopy gradient in a Central Amazonian tropical forest. Insects were sampled over two weeks using 6-m Gressitt-style Malaise traps set at five heights (0 m–32 m–8 m intervals) on a metal tower in a tropical forest north of Manaus, Brazil. The traps contained 37,778 specimens of 18 orders of insects. Using simulation approaches and nonparametric analyses, we interpreted the abundance and richness of insects along this gradient. Diptera, Hymenoptera, and Coleoptera had their greatest abundance at the ground level, whereas Lepidoptera and Hemiptera were more abundant in the upper levels of the canopy. We identified species of 38 of the 56 families of Diptera, finding that 527 out of 856 species (61.6%) were not sampled at the ground level. Mycetophilidae, Tipulidae, and Phoridae were significantly more diverse and/or abundant at the ground level, while Tachinidae, Dolichopodidae, and Lauxaniidae were more diverse or abundant at upper levels. Our study suggests the need for a careful discussion of strategies of tropical forest conservation based on a much more complete understanding of the three-dimensional distribution of its insect diversity.

## Introduction

Tropical forests are the most diverse biome on the planet and also among the most endangered. Habitat loss due to anthropogenic commercial activities threatens the future of the plants and animals that inhabit these ecosystems. To understand and hopefully save these vital, complex environments, it is necessary to understand the biodiversity within in much greater detail. In particular, it is crucial to quantify and document the abundance and species richness of their most species-rich taxa, such as insects. Although challenging, the precise assessment of insect diversity and abundance is a fundamental prerequisite to estimating and mitigating biodiversity loss in tropical forests.

Over the last four decades, studies have uncovered unexpected additional complexity in these forests with the canopy (the leafy crowns of trees) as a vertically stratified ecosystem interconnected with other strata of the forests^1^. Different aspects of canopy ecology and diversity have been studied to date, including climatic gradients within the forest^2^, biomass^3,4^, abundance^5^, alpha diversity^6^, beta diversity^7^, species interactions^8^, biogeochemical cycles^9^, guild structure^10^, long-term succession^11^, community organization^12^, models of vertical distribution^13^, human impact^14^, and other parameters, with a growing number of papers on techniques and methods^15–28^. For example, a study on the island of Borneo^5^ demonstrated that 85% of the variability in arthropod abundance is explained by variability in total leaf area—hence, understory vegetation and canopy contributed disproportionately to the total abundance. In an extensive study of canopy insects in Panama^10^, peaks of abundance at the ground level and in the canopy were found, with most adult arthropods collected either from the soil/litter or from the upper canopy.

Many studies are now available on the diversity of particular insect groups in the canopy. Nevertheless, the underlying questions guiding these studies vary considerably, including the collecting techniques, the level of taxonomic identification, and the core group of insects considered. Some studies approach an array of arthropod groups^29–36^, while most focus on particular groups of lepidopterans^37–47^, beetles^48–53^, hymenopterans^54–58^, orthopterans^59^, collembolans^60,61^, psocopterans^62^ or hemipterans^63^.

Diptera (true flies) are possibly the least-studied of the megadiverse insect orders, and much remains to be discovered about the diversity of the fly fauna in tropical forests. Aside from the heavily researched disease vectors of medical importance (mosquitoes and phlebotomine sand flies), most groups of flies have received much less attention from tropical biologists than more charismatic groups of insects. The number of species of flies described worldwide is about 160,000 and comprise about 10% of all known species of organisms. However, based on projections from mass-sampling projects^64–66^, the number of existing Diptera species is likely much higher. For example, an all-Diptera diversity inventory in Costa Rica estimated about 8000 species in a single two-hectare forest site in Zurquí.

Here, we set out to deepen our understanding of the variability of the insect fauna across a vertical canopy gradient in a Central Amazonian tropical forest. The present study has a two-fold objective. First, we documented the variability of the insect community abundance along a canopy height gradient. Second, we focused our analysis on the Diptera subcomponent of the insect community to understand how this ecologically important and diverse group of insects varies in abundance and species richness at distinct heights of the tropical forest canopy gradient. Published data shows that the insect species composition is not homogeneous across the vertical structure of the forest^67^, so we expected that a more intensive study of flies, in particular, would show considerable variation along the canopy height gradient, similar to that was found for other taxa in other systems^68^.

## Methods

We assessed the vertical structure of insect diversity in a tropical forest using a novel sampling protocol. We used five 6-m long Gressitt-style Malaise traps set at every 8 m from the ground level to slightly above the canopy top at 32 m. The tower (53-m high) is located at the “Estação Experimental de Silvicultura Tropical (EEST)”, km 14 of the road ZF2 (2° 35′ 21″ S, 60° 06′ 55″ W) and belongs to the Instituto Nacional de Pesquisas da Amazônia (INPA), in Manaus, Brazil (briefly referred to here as the ZF2 tower). The tower is located inside a typical Amazonian landscape comprised of ombrophilous dense forest. The canopy reached 40 or even 50 m high due to emergent trees, with a mean canopy height of 28.6 m at the area of the tower^69^. The solid floor at each tower level, which prevents insects from escaping at the lower part of the traps, combined with the trap model used, produced much larger catches of target insects than other canopy sampling methods we have tried.

All traps have biases, and Malaise traps are knowingly much better for collecting active, flying insects such as Diptera and Hymenoptera^70^. We view this bias as a benefit since we are attempting to maximize sampling the diversity of Diptera in our project. We are not aware of any in-depth studies on the differences in catch of the Gressitt-style Malaise traps^71^ used here from those of the more frequently employed Towes-style traps other than the assertion in the original paper that the Gressitt-style traps catch more specimens ^71^. It has been mentioned^72^ a possible drawback to Gressitt-style traps, in that they would be heavy, difficult to place in the field and potentially would not catch low or weakly-flying insects. This latter assertion, to our knowledge, has not been formally tested and does not fit our own observation while sampling with this trap.

Sampling was conducted over a single period of two weeks, during which traps were operated at ground level, 8, 16, 24, and 32 m levels. Insect specimens of each trap were sorted to order and counted; flies were then sorted down to family and counted. Taxonomic specialists for 38 of the sampled fly families identified morphospecies assigned to genera (except for phorids, only assigned to genera). Specialists used different preparation techniques for dealing with their specimens as appropriate (critical-point-drying, slide-mounting, dissection of the terminalia, etc.).

To address our two objectives, we took two analytical approaches. First, to document the variability of the general insect abundance along the vertical gradient, we used a simulation approach to visually understand how the insect orders varied in abundance across trapping locations. Because our data were based on counts, we simulated a dataset following a Poisson distribution using the ‘rpois’ function in R^73^. We established the lambda value (or the rate) as the total number of counts of specimens within megadiverse groups at a trapping location, and we simulated the distribution based on 1000 samples from each height (5000 in total) (Supplementary Appendix A). Therefore, we assumed that the total count from our samples represents the mean of a potential 5000 survey efforts with our approach. We used this approach strictly for visualization purposes to demonstrate how megadiverse insects varied among different levels on the tower.

Second, to understand whether fly abundances varied at distinct heights of the tropical forest canopy gradient, we used nonparametric Kruskal–Wallis tests, with the specimen count (abundance) of a family as the dependent variable and the canopy height as the categorical independent variable. We utilized the complete specimen database from field samples for abundance (total counts,* n* = 8386). When Kruskal–Wallis tests were significant, we computed a multiple comparisons routine using a nonparametric procedure based on relative contrast effects (nparcomp package in R^74^). We evaluated pairwise comparisons among groups using a Bonferroni adjusted alpha value (0.05/10 = 0.005). To compare patterns of abundance across canopy heights with richness, we again used a simulation routine. Because our species richness data were an accumulation of species occurrences across heights, we simulated the variability of species richness if we were to resample traps 5000 times using identical methods to those described for the insect orders. This approach yielded visualizations where, in some cases, species richness was higher than abundance (e.g., Diptera, Tachinidae). We stress that the pattern is because the abundance plots and analyses are based on the mean abundances of Diptera and associated families, whereas the species richness plots are based on total species in the simulated datasets. Thus, the patterns among abundance and richness plots are the important components of the approach. To highlight the variability of insect groups across the five canopy heights, we created box plots using the 'ggpubr' package in R^75^. When discussing the vertical patterns found, we refer to a "peak" when the abundance or species richness at a given level is over 5% higher than adjacent levels (for levels 8 m, 16 m, and 24 m) or at the adjacent superior or inferior level (respectively for samples at the ground level and 32 m); otherwise, we consider a “plateau” across two levels.

## Results

### Objective one—variability of the insect community along a canopy height gradient

**Figure 1:**
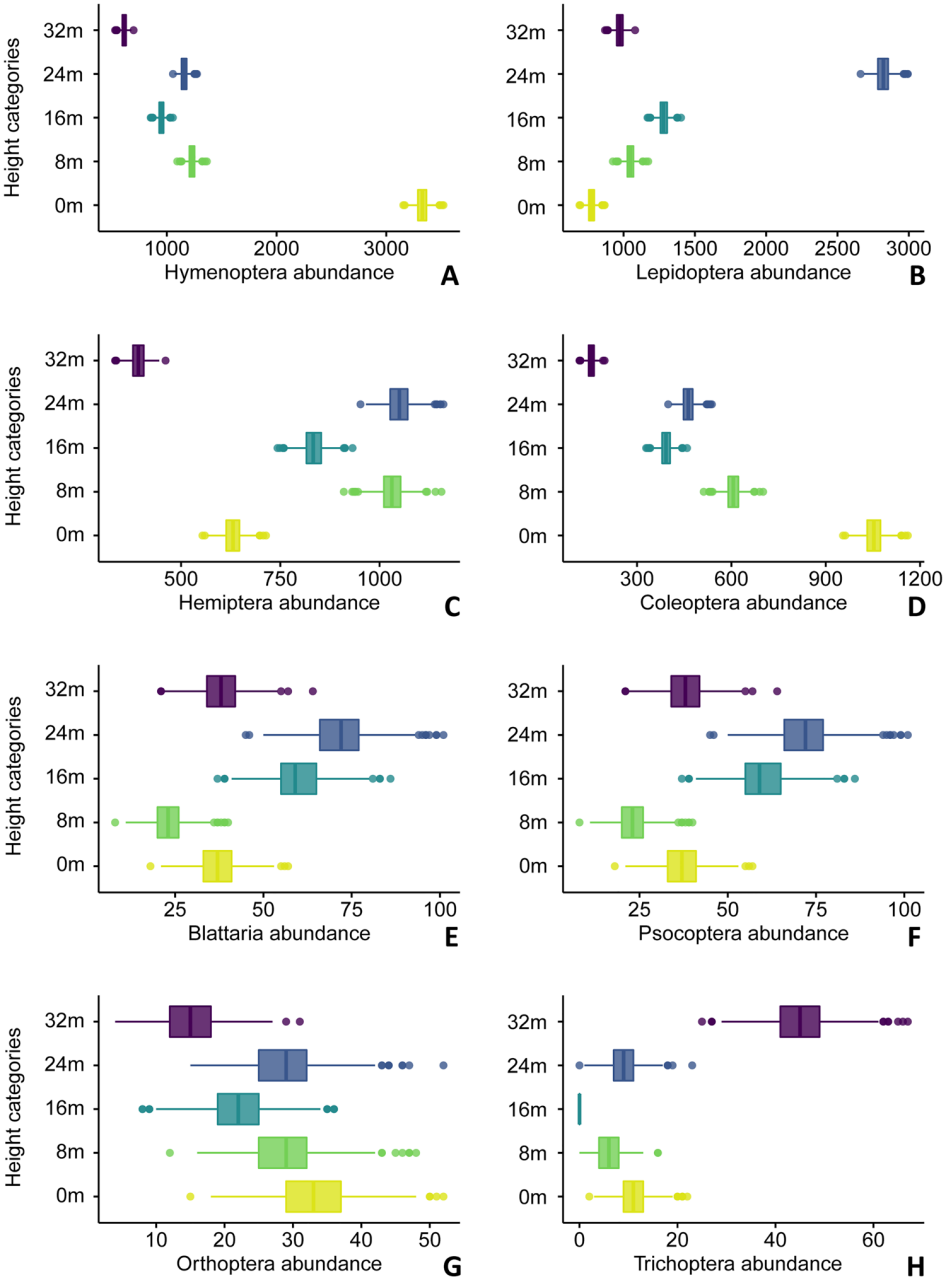
(**A**–**H**) Bar charts of simulated abundance based on the total counts of sampled hexapod orders across the canopy gradient (Diptera in Fig. 2). (**A**) Hymenoptera. (**B**) Lepidoptera. (**C**) Hemiptera. (**D**) Coleoptera. (**E**) Blattaria. (**F**) Psocoptera. (**G**) Orthoptera. (**H**) Trichoptera.

We identified a total of 37,778 insect specimens belonging to 18 orders at the five sampling levels (Supplementary Information Table S1). The most abundant order of insects were the flies (16,600 specimens in the samples), followed by hymenopterans (wasps, bees, and ants, 7279 specimens), moths and butterflies (6899), true bugs (3939), and beetles (2670). The insect orders varied considerably among the trap heights (Fig. 1). Diptera, Hymenoptera, and Coleoptera were more abundant at the ground level and less so at the highest levels of our sample (32 m) (Fig. 1). At the same time, Lepidoptera and Hemiptera were more abundant in the upper levels of the tree canopy (respectively, 24 m and 8–24 m, Fig. 1). For smaller orders, we noticed less clear visual patterns of differences among heights—outside of Trichoptera, which were more abundant above the canopy (32 m), and Blattaria and Psoptera, which appeared to be more abundant in the mid-portions of the canopy (16–24 m) (Fig. 1).

### Objective two—variability of Diptera at distinct heights of the tropical forest canopy gradient

**Figure 2:**
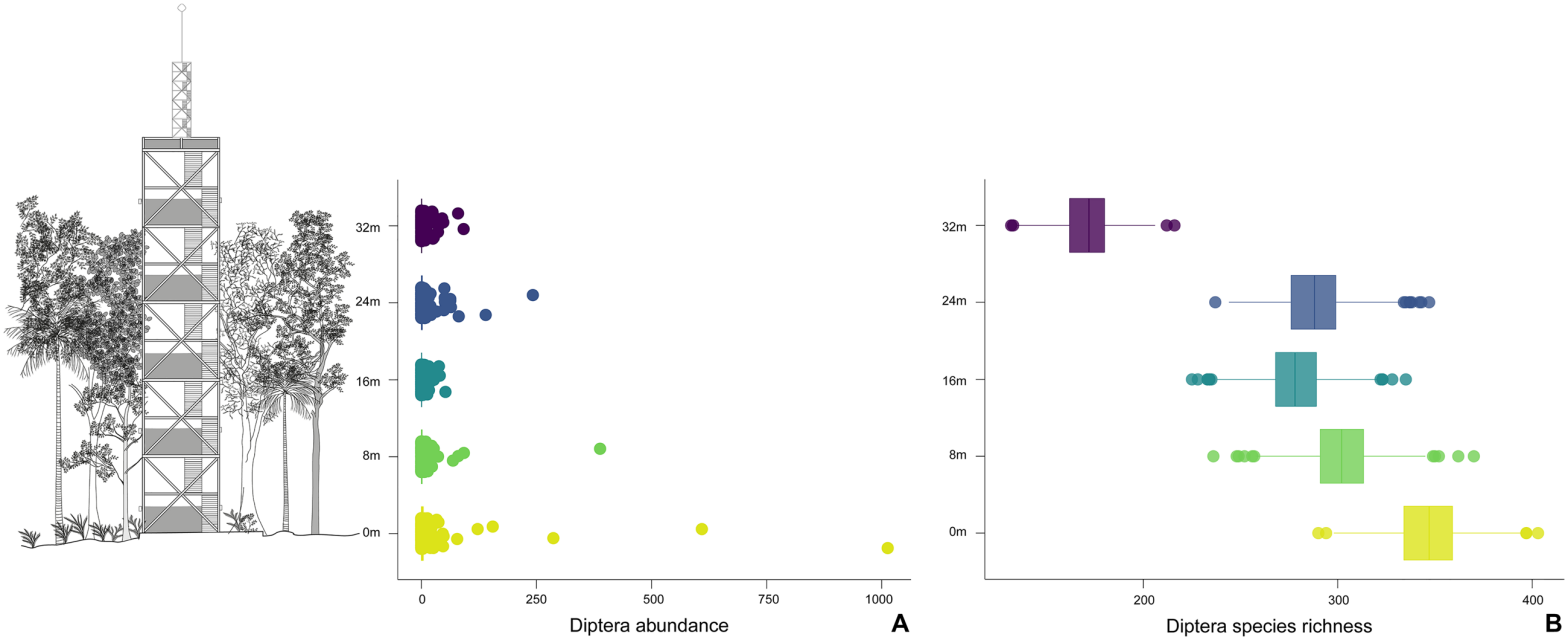
(**A**, **B**) Sketch of a tropical forest and the ZF2 tower, with the distribution of Malaise traps (in grey) set at different heights of the tower. (**A**) Box plots representing the vertical distribution of the mean abundance from the field collection. (**B**) Simulated species richness from the total species accumulation.

The 16,600 Diptera specimens belong to 56 families (Supplementary Information Table S2) (Fig. 2). The most abundant family were the Phoridae (3843 specimens in the samples), followed by Sciaridae (2066), Cecidomyiidae (1977), Psychodidae (1178), Dolichopodidae (935), Chironomidae (880), Tachinidae (833), Mycetophilidae (797), Tipulidae s.l. (738), and Ceratopogonidae (629). A total of 8386 fly specimens of 38 families sampled were identified to morphospecies (or to a genus in the hyperdiverse family Phoridae), belonging to 856 species of 368 genera (Supplementary Information Table S3). Of the families identified to species, the most species-rich was Tachinidae (166 species), followed by Mycetophilidae (101), Tipulidae s.l. (78), Dolichopodidae (71), Drosophilidae (51), Lauxaniidae (46), Muscidae (41), Milichiidae (37), Stratiomyidae (32), and Chloropidae (29). We compiled the cases in which the biology of the larvae or of the adult of the genera sampled was known, providing a sketch of the vertical distribution of guilds of flies (Supplementary Information Table S4). The Supplementary Material Table S5 has the complete dataset of insects identified at each level of the tower.

Overall, dipterans, as an order, were more abundant and species-rich at the ground level (0 m) and less so at the upper canopy (32 m) (*p-value* < 0.001, Fig. 2, Supplementary Appendix A). This pattern was primarily driven by Mycetophilidae (both abundance and richness), Tipulidae (both abundance and richness), and Phoridae (abundance), each of which were significantly more abundant at the 0 m height than at other height categories (*p-value* < 0.001, Supplementary Appendix A). On the other end of the gradient, Tachinidae and Dolichopodidae were significantly more abundant and species-rich at the two highest trap locations (24–32 m) compared with the ground level trap (0 m) (*p-value* = 0.001, Fig. 2). Patterns of species richness generally mirrored those of abundance with two key exceptions. First, Dolichopodidae appears to have incongruence at 16 m between species richness and abundance (Fig. 2). Further, Phoridae genus richness appears greater from 8 to 32 m relative to abundance values, suggesting these flies utilize varying niches at upper levels of the forest canopy with a small population size (Fig. 2).

## Discussion

### Insect order abundance patterns

This study was designed to fill a gap in our knowledge on canopy insect diversity by documenting the variability of the insect fauna across a vertical canopy gradient in a Central Amazonian tropical forest. Our use of the 6 m Gressitt traps was amazingly productive for Diptera and assessed a large number of rare and new flies—such as odiniids, possibly many new genera of phorids, large number of species of lauxaniids, etc. The efficiency of the traps used in this study (as compared to the canopy Malaise traps in the ZADBI project^14,15^) in good proportion seems due to the large interception surface of the 6 m traps with two collector vials and to the fact that the tower platforms provide a floor for the traps, preventing flies from escaping.

Moreover, our sampling approach also combines the discrimination of the fauna at more levels in the forest and a much more extensive taxonomic study in Diptera, depicting in more detail the vertical structure of abundance and species richness. In Panama^10^, ground level ("understory") samples included collecting from 0 to 3 m above the floor, while canopy ("lower canopy") combined the results of all samples from 3 to 35 m above the ground—in our study subdivided, showing notable differences. Finally, their data for Diptera covers just seven families, of which only four had more than ten specimens. Of 188 species of flies that they identified in the tropical forest in Panama, 92 (49%) occur at the ground level, while 120 (64%) occur at the canopy and 50 (27%) above the canopy. Thus, our results are, overall, concordant with the Panama study.

It has been argued that the main driver for spatial and temporal distribution patterns is largely the availability of resources^18^. In our case, this means that the vertical patterns of insect abundance would reflect how different groups explore resources available in the vertical structure of the forest. In our study, the ground-level sample individually has the highest insect abundance. However, over two-thirds of the insect abundance was collected in traps above the ground level. This suggests that the amount of food and other resources for flies at the ground level may be more considerable than at any individual level. Still, the sum of resources (and range of resource types) at all levels above the ground is higher than that at the ground level itself, illustrating the importance of the upper canopy as a distinct set of habitats with varied niches for insects.

The vertical distribution of abundance was not the same across the 18 sampled insect orders (Supplementary Information Table S1). Of 11 orders with more than ten sampled specimens, three have the highest abundance at the ground level, including termites, collembolans, and psocopterans. For each, their abundance decreased away from the ground towards the canopy. Four orders had a peak of abundance at the ground level, with a second peak at 24 m—orthopterans, beetles, hymenopterans, and flies. True bugs (hemipterans) and neuropterans have a 2-peaks abundance pattern (at 8 m and 24 m), while cockroaches and lepidopterans have a 1-peak abundance pattern at the canopy (at 16 m or 24 m). Trichopterans (which have aquatic larvae) were the only group with a peak of abundance at 32 m—probably using the open area above the canopy as an avenue for dispersal. The fact that Blattaria, Hemiptera, Neuroptera, Trichoptera, and Lepidoptera have over 80% of their abundance above the ground level is particularly remarkable.

### Diptera family vertical abundance patterns

The combined abundance of flies at the four higher levels of the tower compared with the abundance at the ground level—1.88—is smaller than the overall proportion of insects between these same levels—2.23 (Supplementary Material Table S2). In other words, the upper canopy is slightly more relevant, in terms of abundance, for some insect orders (such as the largely herbivorous moth and butterfly fauna, and the cockroaches, bugs, and bugs the neuropteran predators, etc.) than it is for flies.

**Figure 3:**
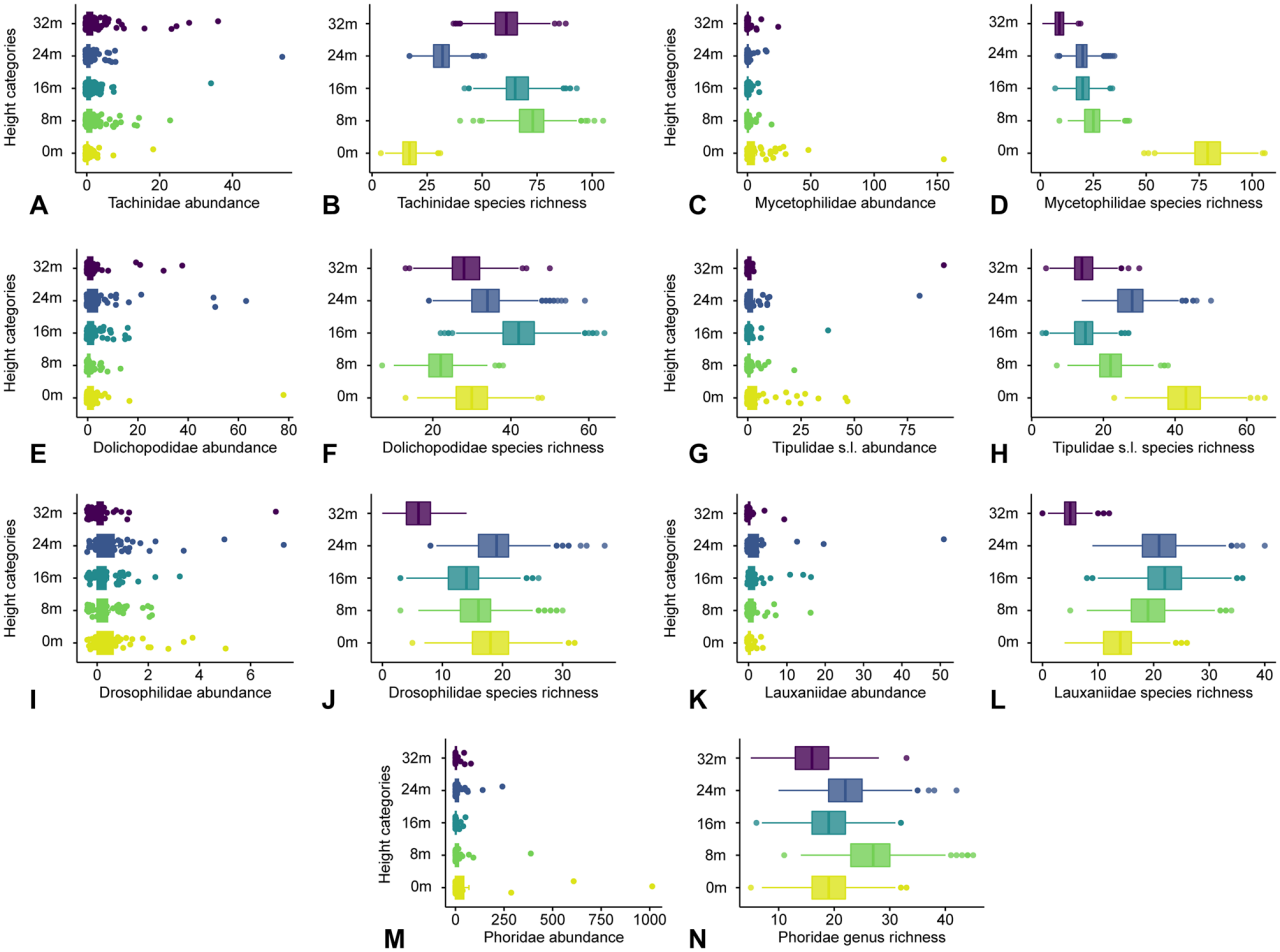
(**A**–**N**) Vertical distribution patterns of the seven most abundant and/or most speciose Diptera families sampled at the ZF2 tower. (**A**, **B**) Tachinidae. (**C**, **D**) Mycetophilidae. (**E**, **F**) Dolichopodidae. (**G**, **H**) Tipulidae s.l. (**I**, **J**) Drosophilidae. (**K**, **L**) Lauxaniidae. (**M**, **N**) Phoridae. The abundance box plots are based on the mean counts from the field collection, whereas the species richness boxplots are simulated data based on the total species accumulation of families (or genus for Phoridae) across the canopy gradient.

Taxon names are hubs of information, connecting different sources of data, including larvae and adult biology. As we deepen the identification from order towards species, we add elements that may help discern and explain patterns found. The assessment of the patterns of vertical abundance of different Diptera families is far more complex (Fig. 3A–N; Supplementary Information Table S2). Only eight among 57 families of flies have a single peak of abundance at the ground level. This includes groups with mycophagous larvae (Mycetophilidae), predatory larvae living in the mud (Tabanidae), adult predators (Asilidae), groups with saprophagous larvae (Micropezidae), and kleptoparasitic flies (Milichiidae). A two-peak pattern in which the ground level is one of the peaks is seen in 16 other fly families. This includes groups that explore very different kinds of resources—e.g., hematophagous flies (Culicidae and Ceratopogonidae), predators (Empididae and Dolichopodidae), but especially phorids (with a wide variety of biologies). A single peak of abundance at 8 m or a 2-peak pattern involving the 8 m level are found in only four families, possibly of flies associated with wood holes and tree trunks or of flies that use the open area between the understory and the canopy to move around in the forest (e.g., the fly parasitoid family Tachinidae). Some other less common patterns include the 2-peak 8/24 m pattern in Chloropidae and the Clusiidae or the 2-peak 0/32 m pattern of the well-known hill-toppers of the family Sarcophagidae (that mate at high places in the landscape). Sciaridae and Cecidomyiidae do not show a very clear pattern and have a more or less regular distribution across the five levels. Overall, the combined abundance of flies at levels 16 m and 24 m (the body of the canopy) is only slightly smaller (33.3%) than the abundance of flies at the ground level (34.7%).

The abundance patterns of the various insect orders and the fly families sampled at the ZF2 tower, in general, concurs with the biology known for these groups. Our study, however, formally measures the relative importance of the canopy fauna at different levels for each of the families, information not available in any previous study. There were some notable surprises: over half of the abundance of crane flies and over one-third of the abundance of fungus-gnats, for example (families connected mainly to resources available at the ground level), were found above the ground. The astonishing 90.0% of abundance of clusiids, 92.6% of lauxaniids, 94.1% of tachinids, and 100% of syrphids at the four higher strata in the forest depict a much more significant proportion of canopy fauna of these families than ever shown before.

### Diptera vertical species-richness patterns

Of the 856 species of flies of 38 families identified to the species level in our study, 329 (38.4%) occurred at the ground level, 188 (22.0%) of which were exclusive to the ground level (Fig. 3A–N; Supplementary Table S3). Similar numbers were obtained in Panama^10^, which compared beta-diversity and found it to be much less significant. One major implication of these numbers is that if insect sampling were made only at ground level, over 60% of the fauna would be missed. In other words, collecting only at the ground level leads to a significant underestimation of the species diversity of a megadiverse order of insects in the Amazon forest.

Ten of the 38 fly families sorted down to species have a species-richness peak at the ground-level, in some cases with a 1-peak pattern (Mycetophilidae and Micropezidae) and some with a second peak in the canopy, either at 16 m or at 24 m—Tipulidae s.l., Tabanidae, Empididae, Drosophilidae, Milichiidae, Stratiomyidae, and Dolichopodidae. There are peaks at 8 m for only five families: Scatopsidae, Chloropidae, Clusiidae, Pipunculidae, and Tachinidae—the latter two of parasitoids. A total of 14 other families have a single peak at the canopy (16 m or 24 m). Of these, Lauxaniidae, Syrphidae, Muscidae, and Odiniidae have a 1-peak pattern, three families, as mentioned, have another peak at 8 m, and eight families also have a peak at the ground level. The only families with a species-richness peak above the canopy are Sarcophagidae and Tachinidae.

### Vulnerability of the fauna

The distinctness and complexity of patterns of the canopy fauna, in some cases with small populations, imply that it may be particularly vulnerable to disturbances by human activity. Climate change already has different impacts on the canopy and its fauna, including loss of biodiversity^1^. Our data strongly corroborates previous reports^10^ that the taxonomic composition of the insect fauna is largely distinct across the vertical structure of the forest and that the canopy harbors a set of species of its own. Selective logging, in this context, removing larger trees, could negatively affect a significant part of the canopy fauna. Further careful studies should be performed to check the effects of selective logging on insect communities and whether the practice is indeed a biodiversity-friendly approach. For the large-scale protection of tropical diversity, there should be core areas of intact forest^76,77^, with all canopy strata conserved, which our results generally support.

Considering the huge pressure on the Amazon primary forest in recent years^78^, in some cases, even with government support, the understanding of its biodiversity becomes a more urgent priority. This cannot be achieved in a "vacuum of data"^79^. This paper fills part of this gap, demonstrating a rich and exclusive canopy fauna and a complex system of vertical patterns of different groups of insects in the Amazon forest. Further, our study raises an array of unanswered questions: the success or failure of conservation actions largely depends on a better understanding of seasonality, phylogeography, abundance, species-richness and guild evolution of the organisms that inhabit them. Concerted action must be made to protect tropical ecosystems—including all canopy levels—against pervasive stressors, such as climate change, deforestation, fragmentation of the landscape, and use of pesticides.

## Supplementary Information

Supplementary Information.

Supplementary Table S1.

Supplementary Table S2.

Supplementary Table S3.

Supplementary Table S4.

Supplementary Table S5.
