# Supplementary material for: Vertical stratification of insect abundance and species richness in an Amazonian tropical forest
Source: Sci Rep. 2022 Feb 2;12:1734. doi: 10.1038/s41598-022-05677-y (PMC8810858; doi:10.1038/s41598-022-05677-y)
Supplement: Supplementary file 1 — Supplementary Information. [file 41598_2022_5677_MOESM1_ESM.html]

Supplementary Material. Appendix A.


# Supplementary Material. Appendix A.

#### Eric M. Wood, Ph.D - ericmwood@calstatela.edu - www.ericmwood.org

---

- This is an R Markdown file associated with the paper “Vertical stratification of insect abundance and species richness in an Amazonian tropical forest” (Amorim et al., Scientific Reports)
- For all queries on the data analyses presented in this document, please contact the author.
- Each code chunk for abundnace has (a) Kruskal-Wallis and multiple comparisons test, (b) means and sd, and (c) boxplots
- Code for the simulated richness data are (a) the code setup and (b) boxplot.

---

# Diptera abundance

```
k1 <-kruskal.test(flies$Abundance ~ as.factor(flies$Group)) 
k1
```

```
## 
##  Kruskal-Wallis rank sum test
## 
## data:  flies$Abundance by as.factor(flies$Group)
## Kruskal-Wallis chi-squared = 74.289, df = 4, p-value = 2.816e-15
```

```
k2 <- nparcomp(Abundance ~ as.factor(Group), data = flies, type = "Tukey")
```

```
## 
##  #------Nonparametric Multiple Comparisons for relative contrast effects-----# 
##  
##  - Alternative Hypothesis:  True relative contrast effect p is not equal to 1/2 
##  - Type of Contrast : Tukey 
##  - Confidence level: 95 % 
##  - Method = Logit - Transformation 
##  - Estimation Method: Pairwise rankings 
##  
##  #---------------------------Interpretation----------------------------------# 
##  p(a,b) > 1/2 : b tends to be larger than a 
##  #---------------------------------------------------------------------------# 
##
```

```
summary(k2)
```

```
## 
##  #------------Nonparametric Multiple Comparisons for relative contrast effects----------# 
##  
##  - Alternative Hypothesis:  True relative contrast effect p is not equal to 1/2 
##  - Estimation Method: Global Pseudo ranks 
##  - Type of Contrast : Tukey 
##  - Confidence Level: 95 % 
##  - Method = Logit - Transformation 
##  
##  - Estimation Method: Pairwise rankings 
##  
##  #---------------------------Interpretation--------------------------------------------# 
##  p(a,b) > 1/2 : b tends to be larger than a 
##  #-------------------------------------------------------------------------------------# 
##  
##  #----Data Info-------------------------------------------------------------------------# 
##        Sample Size
## 1       Eight  867
## 2     Sixteen  867
## 3  Thirty_two  867
## 4 Twenty_Four  867
## 5        Zero  867
## 
##  #----Contrast--------------------------------------------------------------------------# 
##                          Eight Sixteen Thirty_two Twenty_Four Zero
## Sixteen - Eight             -1       1          0           0    0
## Thirty_two - Eight          -1       0          1           0    0
## Twenty_Four - Eight         -1       0          0           1    0
## Zero - Eight                -1       0          0           0    1
## Thirty_two - Sixteen         0      -1          1           0    0
## Twenty_Four - Sixteen        0      -1          0           1    0
## Zero - Sixteen               0      -1          0           0    1
## Twenty_Four - Thirty_two     0       0         -1           1    0
## Zero - Thirty_two            0       0         -1           0    1
## Zero - Twenty_Four           0       0          0          -1    1
## 
##  #----Analysis--------------------------------------------------------------------------# 
##                       Comparison Estimator Lower Upper  Statistic      p.Value
## 1           p( Eight , Sixteen )     0.477 0.446 0.508 -1.9898429 2.707358e-01
## 2        p( Eight , Thirty_two )     0.428 0.399 0.458 -6.5594139 1.544289e-10
## 3       p( Eight , Twenty_Four )     0.482 0.450 0.513 -1.5933997 5.023040e-01
## 4              p( Eight , Zero )     0.523 0.490 0.555  1.8825062 3.269358e-01
## 5      p( Sixteen , Thirty_two )     0.450 0.421 0.479 -4.6693508 1.135792e-05
## 6     p( Sixteen , Twenty_Four )     0.504 0.473 0.535  0.3513046 9.970241e-01
## 7            p( Sixteen , Zero )     0.545 0.513 0.577  3.8502308 7.347897e-04
## 8  p( Thirty_two , Twenty_Four )     0.553 0.524 0.581  4.9222782 1.100492e-05
## 9         p( Thirty_two , Zero )     0.593 0.563 0.622  8.2577133 6.661338e-16
## 10       p( Twenty_Four , Zero )     0.540 0.508 0.572  3.3963012 5.500490e-03
## 
##  #----Overall---------------------------------------------------------------------------# 
##   Quantile      p.Value
## 1 2.720118 6.661338e-16
## 
##  #--------------------------------------------------------------------------------------#
```

```
group_by(flies, Group) %>%
  summarise(
    count = n(),
    mean = mean(Abundance, na.rm = TRUE),
    sd = sd(Abundance, na.rm = TRUE)
  )
```

```
## # A tibble: 5 x 4
##   Group       count  mean    sd
##   <chr>       <int> <dbl> <dbl>
## 1 Eight         867 1.77  14.2 
## 2 Sixteen       867 0.999  3.62
## 3 Thirty_two    867 1.11   5.74
## 4 Twenty_Four   867 2.09  11.7 
## 5 Zero          867 4.19  42.1
```

```
g1 <- ggbarplot(flies,
          x = "Group",
          y = "Abundance",
          add = c("mean_se", "jitter"),
          color = "Group",
          fill = "Group", palette = c("#DCE319FF", "#73D055FF", "#238A8DFF", "#39568CFF", "#440154FF"), alpha=0.5,
          order = c("Zero", "Eight", "Sixteen", "Twenty_Four", "Thirty_two"),
          ylab = "Diptera abundance",
          xlab = "Height categories")

g1 + coord_flip()
```

---

# Tachinidae abundance

```
k1 <-kruskal.test(Tachinidae$Abundance ~ as.factor(Tachinidae$Group)) 
k1
```

```
## 
##  Kruskal-Wallis rank sum test
## 
## data:  Tachinidae$Abundance by as.factor(Tachinidae$Group)
## Kruskal-Wallis chi-squared = 62.272, df = 4, p-value = 9.657e-13
```

```
k2 <- nparcomp(Abundance ~ as.factor(Group), data = Tachinidae, type = "Tukey")
```

```
## 
##  #------Nonparametric Multiple Comparisons for relative contrast effects-----# 
##  
##  - Alternative Hypothesis:  True relative contrast effect p is not equal to 1/2 
##  - Type of Contrast : Tukey 
##  - Confidence level: 95 % 
##  - Method = Logit - Transformation 
##  - Estimation Method: Pairwise rankings 
##  
##  #---------------------------Interpretation----------------------------------# 
##  p(a,b) > 1/2 : b tends to be larger than a 
##  #---------------------------------------------------------------------------# 
##
```

```
summary(k2)
```

```
## 
##  #------------Nonparametric Multiple Comparisons for relative contrast effects----------# 
##  
##  - Alternative Hypothesis:  True relative contrast effect p is not equal to 1/2 
##  - Estimation Method: Global Pseudo ranks 
##  - Type of Contrast : Tukey 
##  - Confidence Level: 95 % 
##  - Method = Logit - Transformation 
##  
##  - Estimation Method: Pairwise rankings 
##  
##  #---------------------------Interpretation--------------------------------------------# 
##  p(a,b) > 1/2 : b tends to be larger than a 
##  #-------------------------------------------------------------------------------------# 
##  
##  #----Data Info-------------------------------------------------------------------------# 
##        Sample Size
## 1       Eight  166
## 2     Sixteen  166
## 3  Thirty_two  166
## 4 Twenty_Four  166
## 5        Zero  166
## 
##  #----Contrast--------------------------------------------------------------------------# 
##                          Eight Sixteen Thirty_two Twenty_Four Zero
## Sixteen - Eight             -1       1          0           0    0
## Thirty_two - Eight          -1       0          1           0    0
## Twenty_Four - Eight         -1       0          0           1    0
## Zero - Eight                -1       0          0           0    1
## Thirty_two - Sixteen         0      -1          1           0    0
## Twenty_Four - Sixteen        0      -1          0           1    0
## Zero - Sixteen               0      -1          0           0    1
## Twenty_Four - Thirty_two     0       0         -1           1    0
## Zero - Thirty_two            0       0         -1           0    1
## Zero - Twenty_Four           0       0          0          -1    1
## 
##  #----Analysis--------------------------------------------------------------------------# 
##                       Comparison Estimator Lower Upper  Statistic      p.Value
## 1           p( Eight , Sixteen )     0.472 0.396 0.549 -0.9953026 8.603156e-01
## 2        p( Eight , Thirty_two )     0.470 0.395 0.546 -1.0758403 8.214569e-01
## 3       p( Eight , Twenty_Four )     0.381 0.315 0.452 -4.5120172 2.415471e-05
## 4              p( Eight , Zero )     0.331 0.272 0.396 -6.8271750 2.564171e-11
## 5      p( Sixteen , Thirty_two )     0.496 0.421 0.571 -0.1435297 9.999786e-01
## 6     p( Sixteen , Twenty_Four )     0.406 0.339 0.476 -3.6646062 2.833029e-03
## 7            p( Sixteen , Zero )     0.354 0.294 0.418 -6.0877798 4.336760e-09
## 8  p( Thirty_two , Twenty_Four )     0.414 0.349 0.483 -3.4089053 4.736690e-03
## 9         p( Thirty_two , Zero )     0.366 0.307 0.428 -5.7331110 3.668978e-08
## 10       p( Twenty_Four , Zero )     0.453 0.401 0.507 -2.3822446 1.175740e-01
## 
##  #----Overall---------------------------------------------------------------------------# 
##   Quantile      p.Value
## 1  2.73107 2.564171e-11
## 
##  #--------------------------------------------------------------------------------------#
```

```
group_by(Tachinidae, Group) %>%
  summarise(
    count = n(),
    mean = mean(Abundance, na.rm = TRUE),
    sd = sd(Abundance, na.rm = TRUE)
  )
```

```
## # A tibble: 5 x 4
##   Group       count  mean    sd
##   <chr>       <int> <dbl> <dbl>
## 1 Eight         166 1.33   2.97
## 2 Sixteen       166 0.952  2.90
## 3 Thirty_two    166 1.60   4.74
## 4 Twenty_Four   166 0.843  4.40
## 5 Zero          166 0.295  1.57
```

```
g2 <- ggbarplot(Tachinidae,
          x = "Group",
          y = "Abundance", 
          add = c("mean_se", "jitter"),
          color = "Group",
          fill = "Group", palette = c("#DCE319FF", "#73D055FF", "#238A8DFF", "#39568CFF", "#440154FF"), alpha=0.5,
          order = c("Zero", "Eight", "Sixteen", "Twenty_Four", "Thirty_two"),
          ylab = "Tachinidae abundance",
          xlab = "Height categories")

g2 + coord_flip()
```

---

# Mycetophilidae abundance

```
k1 <-kruskal.test(Mycetophilidae$Abundance ~ as.factor(Mycetophilidae$Group)) 
k1
```

```
## 
##  Kruskal-Wallis rank sum test
## 
## data:  Mycetophilidae$Abundance by as.factor(Mycetophilidae$Group)
## Kruskal-Wallis chi-squared = 147.37, df = 4, p-value < 2.2e-16
```

```
k2 <- nparcomp(Abundance ~ as.factor(Group), data = Mycetophilidae, type = "Tukey")
```

```
## 
##  #------Nonparametric Multiple Comparisons for relative contrast effects-----# 
##  
##  - Alternative Hypothesis:  True relative contrast effect p is not equal to 1/2 
##  - Type of Contrast : Tukey 
##  - Confidence level: 95 % 
##  - Method = Logit - Transformation 
##  - Estimation Method: Pairwise rankings 
##  
##  #---------------------------Interpretation----------------------------------# 
##  p(a,b) > 1/2 : b tends to be larger than a 
##  #---------------------------------------------------------------------------# 
##
```

```
summary(k2)
```

```
## 
##  #------------Nonparametric Multiple Comparisons for relative contrast effects----------# 
##  
##  - Alternative Hypothesis:  True relative contrast effect p is not equal to 1/2 
##  - Estimation Method: Global Pseudo ranks 
##  - Type of Contrast : Tukey 
##  - Confidence Level: 95 % 
##  - Method = Logit - Transformation 
##  
##  - Estimation Method: Pairwise rankings 
##  
##  #---------------------------Interpretation--------------------------------------------# 
##  p(a,b) > 1/2 : b tends to be larger than a 
##  #-------------------------------------------------------------------------------------# 
##  
##  #----Data Info-------------------------------------------------------------------------# 
##        Sample Size
## 1       Eight  103
## 2     Sixteen  103
## 3  Thirty_two  103
## 4 Twenty_Four  103
## 5        Zero  103
## 
##  #----Contrast--------------------------------------------------------------------------# 
##                          Eight Sixteen Thirty_two Twenty_Four Zero
## Sixteen - Eight             -1       1          0           0    0
## Thirty_two - Eight          -1       0          1           0    0
## Twenty_Four - Eight         -1       0          0           1    0
## Zero - Eight                -1       0          0           0    1
## Thirty_two - Sixteen         0      -1          1           0    0
## Twenty_Four - Sixteen        0      -1          0           1    0
## Zero - Sixteen               0      -1          0           0    1
## Twenty_Four - Thirty_two     0       0         -1           1    0
## Zero - Thirty_two            0       0         -1           0    1
## Zero - Twenty_Four           0       0          0          -1    1
## 
##  #----Analysis--------------------------------------------------------------------------# 
##                       Comparison Estimator Lower Upper  Statistic      p.Value
## 1           p( Eight , Sixteen )     0.472 0.391 0.555 -0.9224584 8.990468e-01
## 2        p( Eight , Thirty_two )     0.420 0.350 0.494 -2.9495095 2.604815e-02
## 3       p( Eight , Twenty_Four )     0.463 0.384 0.544 -1.2550408 7.298225e-01
## 4              p( Eight , Zero )     0.774 0.677 0.848  6.8962697 1.454703e-11
## 5      p( Sixteen , Thirty_two )     0.445 0.378 0.515 -2.1551561 1.987147e-01
## 6     p( Sixteen , Twenty_Four )     0.490 0.413 0.567 -0.3520344 9.985649e-01
## 7            p( Sixteen , Zero )     0.807 0.716 0.874  7.7716850 2.731149e-14
## 8  p( Thirty_two , Twenty_Four )     0.546 0.478 0.612  1.8474204 3.519764e-01
## 9         p( Thirty_two , Zero )     0.844 0.757 0.904  8.3612651 3.330669e-16
## 10       p( Twenty_Four , Zero )     0.815 0.725 0.881  7.9071744 6.550316e-15
## 
##  #----Overall---------------------------------------------------------------------------# 
##   Quantile      p.Value
## 1 2.740842 3.330669e-16
## 
##  #--------------------------------------------------------------------------------------#
```

```
group_by(Mycetophilidae, Group) %>%
  summarise(
    count = n(),
    mean = mean(Abundance, na.rm = TRUE),
    sd = sd(Abundance, na.rm = TRUE)
  )
```

```
## # A tibble: 5 x 4
##   Group       count  mean    sd
##   <chr>       <int> <dbl> <dbl>
## 1 Eight         103 0.757  2.33
## 2 Sixteen       103 0.447  1.33
## 3 Thirty_two    103 0.563  2.76
## 4 Twenty_Four   103 0.592  2.22
## 5 Zero          103 5.38  16.7
```

```
g3 <- ggbarplot(Mycetophilidae,
          x = "Group",
          y = "Abundance", 
          color = "Group",
          add = c("mean_se", "jitter"),
          fill = "Group", palette = c("#DCE319FF", "#73D055FF", "#238A8DFF", "#39568CFF", "#440154FF"), alpha=1,
          order = c("Zero", "Eight", "Sixteen", "Twenty_Four", "Thirty_two"),
          ylab = "Mycetophilidae abundance",
          xlab = "Height categories")

g3 + coord_flip()
```

\*\*\*

# Dolichopodidae abundance

```
k1 <-kruskal.test(Dolichopodidae$Abundance ~ as.factor(Dolichopodidae$Group)) 
k1
```

```
## 
##  Kruskal-Wallis rank sum test
## 
## data:  Dolichopodidae$Abundance by as.factor(Dolichopodidae$Group)
## Kruskal-Wallis chi-squared = 14.277, df = 4, p-value = 0.006462
```

```
k2 <- nparcomp(Abundance ~ as.factor(Group), data = Mycetophilidae, type = "Tukey")
```

```
## 
##  #------Nonparametric Multiple Comparisons for relative contrast effects-----# 
##  
##  - Alternative Hypothesis:  True relative contrast effect p is not equal to 1/2 
##  - Type of Contrast : Tukey 
##  - Confidence level: 95 % 
##  - Method = Logit - Transformation 
##  - Estimation Method: Pairwise rankings 
##  
##  #---------------------------Interpretation----------------------------------# 
##  p(a,b) > 1/2 : b tends to be larger than a 
##  #---------------------------------------------------------------------------# 
##
```

```
summary(k2)
```

```
## 
##  #------------Nonparametric Multiple Comparisons for relative contrast effects----------# 
##  
##  - Alternative Hypothesis:  True relative contrast effect p is not equal to 1/2 
##  - Estimation Method: Global Pseudo ranks 
##  - Type of Contrast : Tukey 
##  - Confidence Level: 95 % 
##  - Method = Logit - Transformation 
##  
##  - Estimation Method: Pairwise rankings 
##  
##  #---------------------------Interpretation--------------------------------------------# 
##  p(a,b) > 1/2 : b tends to be larger than a 
##  #-------------------------------------------------------------------------------------# 
##  
##  #----Data Info-------------------------------------------------------------------------# 
##        Sample Size
## 1       Eight  103
## 2     Sixteen  103
## 3  Thirty_two  103
## 4 Twenty_Four  103
## 5        Zero  103
## 
##  #----Contrast--------------------------------------------------------------------------# 
##                          Eight Sixteen Thirty_two Twenty_Four Zero
## Sixteen - Eight             -1       1          0           0    0
## Thirty_two - Eight          -1       0          1           0    0
## Twenty_Four - Eight         -1       0          0           1    0
## Zero - Eight                -1       0          0           0    1
## Thirty_two - Sixteen         0      -1          1           0    0
## Twenty_Four - Sixteen        0      -1          0           1    0
## Zero - Sixteen               0      -1          0           0    1
## Twenty_Four - Thirty_two     0       0         -1           1    0
## Zero - Thirty_two            0       0         -1           0    1
## Zero - Twenty_Four           0       0          0          -1    1
## 
##  #----Analysis--------------------------------------------------------------------------# 
##                       Comparison Estimator Lower Upper  Statistic      p.Value
## 1           p( Eight , Sixteen )     0.472 0.391 0.555 -0.9224584 8.990468e-01
## 2        p( Eight , Thirty_two )     0.420 0.350 0.494 -2.9495095 2.604815e-02
## 3       p( Eight , Twenty_Four )     0.463 0.384 0.544 -1.2550408 7.298225e-01
## 4              p( Eight , Zero )     0.774 0.677 0.848  6.8962697 1.454703e-11
## 5      p( Sixteen , Thirty_two )     0.445 0.378 0.515 -2.1551561 1.987147e-01
## 6     p( Sixteen , Twenty_Four )     0.490 0.413 0.567 -0.3520344 9.985649e-01
## 7            p( Sixteen , Zero )     0.807 0.716 0.874  7.7716850 2.731149e-14
## 8  p( Thirty_two , Twenty_Four )     0.546 0.478 0.612  1.8474204 3.519764e-01
## 9         p( Thirty_two , Zero )     0.844 0.757 0.904  8.3612651 3.330669e-16
## 10       p( Twenty_Four , Zero )     0.815 0.725 0.881  7.9071744 6.550316e-15
## 
##  #----Overall---------------------------------------------------------------------------# 
##   Quantile      p.Value
## 1 2.740842 3.330669e-16
## 
##  #--------------------------------------------------------------------------------------#
```

```
group_by(Dolichopodidae, Group) %>%
  summarise(
    count = n(),
    mean = mean(Abundance, na.rm = TRUE),
    sd = sd(Abundance, na.rm = TRUE)
  )
```

```
## # A tibble: 5 x 4
##   Group       count  mean    sd
##   <chr>       <int> <dbl> <dbl>
## 1 Eight          81 0.926  2.09
## 2 Sixteen        81 2.37   3.89
## 3 Thirty_two     81 2.05   6.13
## 4 Twenty_Four    81 3.94  10.8 
## 5 Zero           81 2.26   8.86
```

```
g4 <- ggbarplot(Dolichopodidae,
          x = "Group",
          y = "Abundance", 
          add = c("mean_se", "jitter"),
          color = "Group",
          fill = "Group", palette = c("#DCE319FF", "#73D055FF", "#238A8DFF", "#39568CFF", "#440154FF"), alpha=0.5,
          order = c("Zero", "Eight", "Sixteen", "Twenty_Four", "Thirty_two"),
          ylab = "Dolichopodidae abundance",
          xlab = "Height categories")

g4 + coord_flip()
```

---

# Tipulidae s.l. abundance

```
k1 <-kruskal.test(Tipulidae$Abundance ~ as.factor(Tipulidae$Group)) 
k1
```

```
## 
##  Kruskal-Wallis rank sum test
## 
## data:  Tipulidae$Abundance by as.factor(Tipulidae$Group)
## Kruskal-Wallis chi-squared = 35.707, df = 4, p-value = 3.325e-07
```

```
k2 <- nparcomp(Abundance ~ as.factor(Group), data = Mycetophilidae, type = "Tukey")
```

```
## 
##  #------Nonparametric Multiple Comparisons for relative contrast effects-----# 
##  
##  - Alternative Hypothesis:  True relative contrast effect p is not equal to 1/2 
##  - Type of Contrast : Tukey 
##  - Confidence level: 95 % 
##  - Method = Logit - Transformation 
##  - Estimation Method: Pairwise rankings 
##  
##  #---------------------------Interpretation----------------------------------# 
##  p(a,b) > 1/2 : b tends to be larger than a 
##  #---------------------------------------------------------------------------# 
##
```

```
summary(k2)
```

```
## 
##  #------------Nonparametric Multiple Comparisons for relative contrast effects----------# 
##  
##  - Alternative Hypothesis:  True relative contrast effect p is not equal to 1/2 
##  - Estimation Method: Global Pseudo ranks 
##  - Type of Contrast : Tukey 
##  - Confidence Level: 95 % 
##  - Method = Logit - Transformation 
##  
##  - Estimation Method: Pairwise rankings 
##  
##  #---------------------------Interpretation--------------------------------------------# 
##  p(a,b) > 1/2 : b tends to be larger than a 
##  #-------------------------------------------------------------------------------------# 
##  
##  #----Data Info-------------------------------------------------------------------------# 
##        Sample Size
## 1       Eight  103
## 2     Sixteen  103
## 3  Thirty_two  103
## 4 Twenty_Four  103
## 5        Zero  103
## 
##  #----Contrast--------------------------------------------------------------------------# 
##                          Eight Sixteen Thirty_two Twenty_Four Zero
## Sixteen - Eight             -1       1          0           0    0
## Thirty_two - Eight          -1       0          1           0    0
## Twenty_Four - Eight         -1       0          0           1    0
## Zero - Eight                -1       0          0           0    1
## Thirty_two - Sixteen         0      -1          1           0    0
## Twenty_Four - Sixteen        0      -1          0           1    0
## Zero - Sixteen               0      -1          0           0    1
## Twenty_Four - Thirty_two     0       0         -1           1    0
## Zero - Thirty_two            0       0         -1           0    1
## Zero - Twenty_Four           0       0          0          -1    1
## 
##  #----Analysis--------------------------------------------------------------------------# 
##                       Comparison Estimator Lower Upper  Statistic      p.Value
## 1           p( Eight , Sixteen )     0.472 0.391 0.555 -0.9224584 8.990468e-01
## 2        p( Eight , Thirty_two )     0.420 0.350 0.494 -2.9495095 2.604815e-02
## 3       p( Eight , Twenty_Four )     0.463 0.384 0.544 -1.2550408 7.298225e-01
## 4              p( Eight , Zero )     0.774 0.677 0.848  6.8962697 1.454703e-11
## 5      p( Sixteen , Thirty_two )     0.445 0.378 0.515 -2.1551561 1.987147e-01
## 6     p( Sixteen , Twenty_Four )     0.490 0.413 0.567 -0.3520344 9.985649e-01
## 7            p( Sixteen , Zero )     0.807 0.716 0.874  7.7716850 2.731149e-14
## 8  p( Thirty_two , Twenty_Four )     0.546 0.478 0.612  1.8474204 3.519764e-01
## 9         p( Thirty_two , Zero )     0.844 0.757 0.904  8.3612651 3.330669e-16
## 10       p( Twenty_Four , Zero )     0.815 0.725 0.881  7.9071744 6.550316e-15
## 
##  #----Overall---------------------------------------------------------------------------# 
##   Quantile      p.Value
## 1 2.740842 3.330669e-16
## 
##  #--------------------------------------------------------------------------------------#
```

```
group_by(Tipulidae, Group) %>%
  summarise(
    count = n(),
    mean = mean(Abundance, na.rm = TRUE),
    sd = sd(Abundance, na.rm = TRUE)
  )
```

```
## # A tibble: 5 x 4
##   Group       count  mean    sd
##   <chr>       <int> <dbl> <dbl>
## 1 Eight          78 1      2.99
## 2 Sixteen        78 0.846  4.39
## 3 Thirty_two     78 1.45  10.4 
## 4 Twenty_Four    78 2.14   9.35
## 5 Zero           78 4.12   9.59
```

```
g5 <- ggbarplot(Tipulidae,
          x = "Group",
          y = "Abundance", 
          add = c("mean_se", "jitter"),
          color = "Group",
          fill = "Group", palette = c("#DCE319FF", "#73D055FF", "#238A8DFF", "#39568CFF", "#440154FF"), alpha=0.5,
          order = c("Zero", "Eight", "Sixteen", "Twenty_Four", "Thirty_two"),
          ylab = "Tipulidae abundance",
          xlab = "Height categories")

g5 + coord_flip()
```

---

# Drosophilidae abundance

```
k1 <-kruskal.test(Drosophilidae$Abundance ~ as.factor(Drosophilidae$Group)) 
k1
```

```
## 
##  Kruskal-Wallis rank sum test
## 
## data:  Drosophilidae$Abundance by as.factor(Drosophilidae$Group)
## Kruskal-Wallis chi-squared = 10.748, df = 4, p-value = 0.02955
```

```
k2 <- nparcomp(Abundance ~ as.factor(Group), data = Mycetophilidae, type = "Tukey")
```

```
## 
##  #------Nonparametric Multiple Comparisons for relative contrast effects-----# 
##  
##  - Alternative Hypothesis:  True relative contrast effect p is not equal to 1/2 
##  - Type of Contrast : Tukey 
##  - Confidence level: 95 % 
##  - Method = Logit - Transformation 
##  - Estimation Method: Pairwise rankings 
##  
##  #---------------------------Interpretation----------------------------------# 
##  p(a,b) > 1/2 : b tends to be larger than a 
##  #---------------------------------------------------------------------------# 
##
```

```
summary(k2)
```

```
## 
##  #------------Nonparametric Multiple Comparisons for relative contrast effects----------# 
##  
##  - Alternative Hypothesis:  True relative contrast effect p is not equal to 1/2 
##  - Estimation Method: Global Pseudo ranks 
##  - Type of Contrast : Tukey 
##  - Confidence Level: 95 % 
##  - Method = Logit - Transformation 
##  
##  - Estimation Method: Pairwise rankings 
##  
##  #---------------------------Interpretation--------------------------------------------# 
##  p(a,b) > 1/2 : b tends to be larger than a 
##  #-------------------------------------------------------------------------------------# 
##  
##  #----Data Info-------------------------------------------------------------------------# 
##        Sample Size
## 1       Eight  103
## 2     Sixteen  103
## 3  Thirty_two  103
## 4 Twenty_Four  103
## 5        Zero  103
## 
##  #----Contrast--------------------------------------------------------------------------# 
##                          Eight Sixteen Thirty_two Twenty_Four Zero
## Sixteen - Eight             -1       1          0           0    0
## Thirty_two - Eight          -1       0          1           0    0
## Twenty_Four - Eight         -1       0          0           1    0
## Zero - Eight                -1       0          0           0    1
## Thirty_two - Sixteen         0      -1          1           0    0
## Twenty_Four - Sixteen        0      -1          0           1    0
## Zero - Sixteen               0      -1          0           0    1
## Twenty_Four - Thirty_two     0       0         -1           1    0
## Zero - Thirty_two            0       0         -1           0    1
## Zero - Twenty_Four           0       0          0          -1    1
## 
##  #----Analysis--------------------------------------------------------------------------# 
##                       Comparison Estimator Lower Upper  Statistic      p.Value
## 1           p( Eight , Sixteen )     0.472 0.391 0.555 -0.9224584 8.990468e-01
## 2        p( Eight , Thirty_two )     0.420 0.350 0.494 -2.9495095 2.604815e-02
## 3       p( Eight , Twenty_Four )     0.463 0.384 0.544 -1.2550408 7.298225e-01
## 4              p( Eight , Zero )     0.774 0.677 0.848  6.8962697 1.454703e-11
## 5      p( Sixteen , Thirty_two )     0.445 0.378 0.515 -2.1551561 1.987147e-01
## 6     p( Sixteen , Twenty_Four )     0.490 0.413 0.567 -0.3520344 9.985649e-01
## 7            p( Sixteen , Zero )     0.807 0.716 0.874  7.7716850 2.731149e-14
## 8  p( Thirty_two , Twenty_Four )     0.546 0.478 0.612  1.8474204 3.519764e-01
## 9         p( Thirty_two , Zero )     0.844 0.757 0.904  8.3612651 3.330669e-16
## 10       p( Twenty_Four , Zero )     0.815 0.725 0.881  7.9071744 6.550316e-15
## 
##  #----Overall---------------------------------------------------------------------------# 
##   Quantile      p.Value
## 1 2.740842 3.330669e-16
## 
##  #--------------------------------------------------------------------------------------#
```

```
group_by(Drosophilidae, Group) %>%
  summarise(
    count = n(),
    mean = mean(Abundance, na.rm = TRUE),
    sd = sd(Abundance, na.rm = TRUE)
  )
```

```
## # A tibble: 5 x 4
##   Group       count  mean    sd
##   <chr>       <int> <dbl> <dbl>
## 1 Eight          51 0.412 0.669
## 2 Sixteen        51 0.353 0.658
## 3 Thirty_two     51 0.235 1.01 
## 4 Twenty_Four    51 0.667 1.31 
## 5 Zero           51 0.627 1.11
```

```
g6 <- ggbarplot(Drosophilidae,
          x = "Group",
          y = "Abundance", 
          add = c("mean_se", "jitter"),
          color = "Group",
          fill = "Group", palette = c("#DCE319FF", "#73D055FF", "#238A8DFF", "#39568CFF", "#440154FF"), alpha=0.5,
          order = c("Zero", "Eight", "Sixteen", "Twenty_Four", "Thirty_two"),
          ylab = "Drosophilidae abundance",
          xlab = "Height categories")

g6 + coord_flip()
```

---

# Lauxaniidae abundance

```
k1 <-kruskal.test(Lauxaniidae$Abundance ~ as.factor(Lauxaniidae$Group)) 
k1
```

```
## 
##  Kruskal-Wallis rank sum test
## 
## data:  Lauxaniidae$Abundance by as.factor(Lauxaniidae$Group)
## Kruskal-Wallis chi-squared = 19.153, df = 4, p-value = 0.0007332
```

```
k2 <- nparcomp(Abundance ~ as.factor(Group), data = Mycetophilidae, type = "Tukey")
```

```
## 
##  #------Nonparametric Multiple Comparisons for relative contrast effects-----# 
##  
##  - Alternative Hypothesis:  True relative contrast effect p is not equal to 1/2 
##  - Type of Contrast : Tukey 
##  - Confidence level: 95 % 
##  - Method = Logit - Transformation 
##  - Estimation Method: Pairwise rankings 
##  
##  #---------------------------Interpretation----------------------------------# 
##  p(a,b) > 1/2 : b tends to be larger than a 
##  #---------------------------------------------------------------------------# 
##
```

```
summary(k2)
```

```
## 
##  #------------Nonparametric Multiple Comparisons for relative contrast effects----------# 
##  
##  - Alternative Hypothesis:  True relative contrast effect p is not equal to 1/2 
##  - Estimation Method: Global Pseudo ranks 
##  - Type of Contrast : Tukey 
##  - Confidence Level: 95 % 
##  - Method = Logit - Transformation 
##  
##  - Estimation Method: Pairwise rankings 
##  
##  #---------------------------Interpretation--------------------------------------------# 
##  p(a,b) > 1/2 : b tends to be larger than a 
##  #-------------------------------------------------------------------------------------# 
##  
##  #----Data Info-------------------------------------------------------------------------# 
##        Sample Size
## 1       Eight  103
## 2     Sixteen  103
## 3  Thirty_two  103
## 4 Twenty_Four  103
## 5        Zero  103
## 
##  #----Contrast--------------------------------------------------------------------------# 
##                          Eight Sixteen Thirty_two Twenty_Four Zero
## Sixteen - Eight             -1       1          0           0    0
## Thirty_two - Eight          -1       0          1           0    0
## Twenty_Four - Eight         -1       0          0           1    0
## Zero - Eight                -1       0          0           0    1
## Thirty_two - Sixteen         0      -1          1           0    0
## Twenty_Four - Sixteen        0      -1          0           1    0
## Zero - Sixteen               0      -1          0           0    1
## Twenty_Four - Thirty_two     0       0         -1           1    0
## Zero - Thirty_two            0       0         -1           0    1
## Zero - Twenty_Four           0       0          0          -1    1
## 
##  #----Analysis--------------------------------------------------------------------------# 
##                       Comparison Estimator Lower Upper  Statistic      p.Value
## 1           p( Eight , Sixteen )     0.472 0.391 0.555 -0.9224584 8.990468e-01
## 2        p( Eight , Thirty_two )     0.420 0.350 0.494 -2.9495095 2.604815e-02
## 3       p( Eight , Twenty_Four )     0.463 0.384 0.544 -1.2550408 7.298225e-01
## 4              p( Eight , Zero )     0.774 0.677 0.848  6.8962697 1.454703e-11
## 5      p( Sixteen , Thirty_two )     0.445 0.378 0.515 -2.1551561 1.987147e-01
## 6     p( Sixteen , Twenty_Four )     0.490 0.413 0.567 -0.3520344 9.985649e-01
## 7            p( Sixteen , Zero )     0.807 0.716 0.874  7.7716850 2.731149e-14
## 8  p( Thirty_two , Twenty_Four )     0.546 0.478 0.612  1.8474204 3.519764e-01
## 9         p( Thirty_two , Zero )     0.844 0.757 0.904  8.3612651 3.330669e-16
## 10       p( Twenty_Four , Zero )     0.815 0.725 0.881  7.9071744 6.550316e-15
## 
##  #----Overall---------------------------------------------------------------------------# 
##   Quantile      p.Value
## 1 2.740842 3.330669e-16
## 
##  #--------------------------------------------------------------------------------------#
```

```
group_by(Lauxaniidae, Group) %>%
  summarise(
    count = n(),
    mean = mean(Abundance, na.rm = TRUE),
    sd = sd(Abundance, na.rm = TRUE)
  )
```

```
## # A tibble: 5 x 4
##   Group       count  mean    sd
##   <chr>       <int> <dbl> <dbl>
## 1 Eight          46 1.17  2.78 
## 2 Sixteen        46 1.59  3.47 
## 3 Thirty_two     46 0.348 1.45 
## 4 Twenty_Four    46 2.57  8.09 
## 5 Zero           46 0.457 0.862
```

```
g7 <- ggbarplot(Lauxaniidae,
          x = "Group",
          y = "Abundance", 
          add = c("mean_se", "jitter"),
          color = "Group",
          fill = "Group", palette = c("#DCE319FF", "#73D055FF", "#238A8DFF", "#39568CFF", "#440154FF"), alpha=0.5,
          order = c("Zero", "Eight", "Sixteen", "Twenty_Four", "Thirty_two"),
          ylab = "Lauxaniidae abundance",
          xlab = "Height categories")

g7 + coord_flip()
```

---

# Phoridae abundance

```
k1 <-kruskal.test(Phoridae$Abundance ~ as.factor(Phoridae$Group)) 
k1
```

```
## 
##  Kruskal-Wallis rank sum test
## 
## data:  Phoridae$Abundance by as.factor(Phoridae$Group)
## Kruskal-Wallis chi-squared = 6.1221, df = 4, p-value = 0.1902
```

```
k2 <- nparcomp(Abundance ~ as.factor(Group), data = Mycetophilidae, type = "Tukey")
```

```
## 
##  #------Nonparametric Multiple Comparisons for relative contrast effects-----# 
##  
##  - Alternative Hypothesis:  True relative contrast effect p is not equal to 1/2 
##  - Type of Contrast : Tukey 
##  - Confidence level: 95 % 
##  - Method = Logit - Transformation 
##  - Estimation Method: Pairwise rankings 
##  
##  #---------------------------Interpretation----------------------------------# 
##  p(a,b) > 1/2 : b tends to be larger than a 
##  #---------------------------------------------------------------------------# 
##
```

```
summary(k2)
```

```
## 
##  #------------Nonparametric Multiple Comparisons for relative contrast effects----------# 
##  
##  - Alternative Hypothesis:  True relative contrast effect p is not equal to 1/2 
##  - Estimation Method: Global Pseudo ranks 
##  - Type of Contrast : Tukey 
##  - Confidence Level: 95 % 
##  - Method = Logit - Transformation 
##  
##  - Estimation Method: Pairwise rankings 
##  
##  #---------------------------Interpretation--------------------------------------------# 
##  p(a,b) > 1/2 : b tends to be larger than a 
##  #-------------------------------------------------------------------------------------# 
##  
##  #----Data Info-------------------------------------------------------------------------# 
##        Sample Size
## 1       Eight  103
## 2     Sixteen  103
## 3  Thirty_two  103
## 4 Twenty_Four  103
## 5        Zero  103
## 
##  #----Contrast--------------------------------------------------------------------------# 
##                          Eight Sixteen Thirty_two Twenty_Four Zero
## Sixteen - Eight             -1       1          0           0    0
## Thirty_two - Eight          -1       0          1           0    0
## Twenty_Four - Eight         -1       0          0           1    0
## Zero - Eight                -1       0          0           0    1
## Thirty_two - Sixteen         0      -1          1           0    0
## Twenty_Four - Sixteen        0      -1          0           1    0
## Zero - Sixteen               0      -1          0           0    1
## Twenty_Four - Thirty_two     0       0         -1           1    0
## Zero - Thirty_two            0       0         -1           0    1
## Zero - Twenty_Four           0       0          0          -1    1
## 
##  #----Analysis--------------------------------------------------------------------------# 
##                       Comparison Estimator Lower Upper  Statistic      p.Value
## 1           p( Eight , Sixteen )     0.472 0.391 0.555 -0.9224584 8.990468e-01
## 2        p( Eight , Thirty_two )     0.420 0.350 0.494 -2.9495095 2.604815e-02
## 3       p( Eight , Twenty_Four )     0.463 0.384 0.544 -1.2550408 7.298225e-01
## 4              p( Eight , Zero )     0.774 0.677 0.848  6.8962697 1.454703e-11
## 5      p( Sixteen , Thirty_two )     0.445 0.378 0.515 -2.1551561 1.987147e-01
## 6     p( Sixteen , Twenty_Four )     0.490 0.413 0.567 -0.3520344 9.985649e-01
## 7            p( Sixteen , Zero )     0.807 0.716 0.874  7.7716850 2.731149e-14
## 8  p( Thirty_two , Twenty_Four )     0.546 0.478 0.612  1.8474204 3.519764e-01
## 9         p( Thirty_two , Zero )     0.844 0.757 0.904  8.3612651 3.330669e-16
## 10       p( Twenty_Four , Zero )     0.815 0.725 0.881  7.9071744 6.550316e-15
## 
##  #----Overall---------------------------------------------------------------------------# 
##   Quantile      p.Value
## 1 2.740842 3.330669e-16
## 
##  #--------------------------------------------------------------------------------------#
```

```
group_by(Phoridae, Group) %>%
  summarise(
    count = n(),
    mean = mean(Abundance, na.rm = TRUE),
    sd = sd(Abundance, na.rm = TRUE)
  )
```

```
## # A tibble: 5 x 4
##   Group       count  mean    sd
##   <chr>       <int> <dbl> <dbl>
## 1 Eight          46 15.5   58.8
## 2 Sixteen        46  3.85  10.5
## 3 Thirty_two     46  5.20  15.3
## 4 Twenty_Four    46 15.7   42.6
## 5 Zero           46 43.2  176.
```

```
g8 <- ggbarplot(Phoridae,
          x = "Group",
          y = "Abundance", 
          add = c("mean_se", "jitter"),
          color = "Group",
          fill = "Group", palette = c("#DCE319FF", "#73D055FF", "#238A8DFF", "#39568CFF", "#440154FF"), alpha=0.5,
          order = c("Zero", "Eight", "Sixteen", "Twenty_Four", "Thirty_two"),
          ylab = "Phoridae abundance",
          xlab = "Height categories")

g8 + coord_flip()
```

---

# Diptera richness

```
height0  <- rpois(1000, 347)
height8  <- rpois(1000, 303)
height16 <- rpois(1000, 278)
height24 <- rpois(1000, 288)
height32 <- rpois(1000, 171)


height0[height0   < 0] <- 0
height8[height8   < 0] <- 0
height16[height16 < 0] <- 0
height24[height24 < 0] <- 0
height32[height32 < 0] <- 0


HT_IZ <- c(height0,height8,height16,height24,height32)

Heights<-rep(seq(1,1000),5)

Groups <- c(rep("height0",1000), rep("height8",1000), rep("height16",1000), rep("height24",1000), rep("height32",1000))

AllData <- data.frame(HT_IZ,Heights,Groups)


g9 <- ggboxplot(AllData,
                x = "Groups",
                y = "HT_IZ", 
                color = "Groups",
                fill = "Groups", palette = c("#DCE319FF", "#73D055FF", "#238A8DFF", "#39568CFF", "#440154FF"), alpha=0.8,
                order = c("height0", "height8", "height16", "height24", "height32"),
                ylab = "Diptera richness",
                xlab = "Height categories")

g9 + coord_flip()
```

---

# Tachinidae richness

```
height0  <- rpois(1000, 17)
height8  <- rpois(1000, 73)
height16 <- rpois(1000, 66)
height24 <- rpois(1000, 32)
height32 <- rpois(1000, 61)

HT_IZ <- c(height0,height8,height16,height24,height32)

Heights<-rep(seq(1,1000),5)

Groups <- c(rep("height0",1000), rep("height8",1000), rep("height16",1000), rep("height24",1000), rep("height32",1000))

AllData <- data.frame(HT_IZ,Heights,Groups)


g10 <- ggboxplot(AllData,
                x = "Groups",
                y = "HT_IZ", 
                color = "Groups",
                fill = "Groups", palette = c("#DCE319FF", "#73D055FF", "#238A8DFF", "#39568CFF", "#440154FF"), alpha=0.8,
                order = c("height0", "height8", "height16", "height24", "height32"),
                ylab = "Tachinidae richness",
                xlab = "Height categories")

g10 + coord_flip()
```

---

# Mycetophilidae richness

```
height0  <- rpois(1000, 79)
height8  <- rpois(1000, 25)
height16 <- rpois(1000, 20)
height24 <- rpois(1000, 20)
height32 <- rpois(1000, 9)

HT_IZ <- c(height0,height8,height16,height24,height32)

Heights<-rep(seq(1,1000),5)

Groups <- c(rep("height0",1000), rep("height8",1000), rep("height16",1000), rep("height24",1000), rep("height32",1000))

AllData <- data.frame(HT_IZ,Heights,Groups)


g11 <- ggboxplot(AllData,
                x = "Groups",
                y = "HT_IZ", 
                color = "Groups",
                fill = "Groups", palette = c("#DCE319FF", "#73D055FF", "#238A8DFF", "#39568CFF", "#440154FF"), alpha=0.8,
                order = c("height0", "height8", "height16", "height24", "height32"),
                ylab = "Mycetophilidae richness",
                xlab = "Height categories")

g11 + coord_flip()
```

---

# Dolichopodidae richness

```
height0  <- rpois(1000, 30)
height8  <- rpois(1000, 22)
height16 <- rpois(1000, 42)
height24 <- rpois(1000, 34)
height32 <- rpois(1000, 28)

HT_IZ <- c(height0,height8,height16,height24,height32)

Heights<-rep(seq(1,1000),5)

Groups <- c(rep("height0",1000), rep("height8",1000), rep("height16",1000), rep("height24",1000), rep("height32",1000))

AllData <- data.frame(HT_IZ,Heights,Groups)


g12 <- ggboxplot(AllData,
                x = "Groups",
                y = "HT_IZ", 
                color = "Groups",
                fill = "Groups", palette = c("#DCE319FF", "#73D055FF", "#238A8DFF", "#39568CFF", "#440154FF"), alpha=0.8,
                order = c("height0", "height8", "height16", "height24", "height32"),
                ylab = "Dolichopodidae richness",
                xlab = "Height categories")

g12 + coord_flip()
```

---

# Tipulidae s.l. richness

```
height0  <- rpois(1000, 43)
height8  <- rpois(1000, 22)
height16 <- rpois(1000, 15)
height24 <- rpois(1000, 28)
height32 <- rpois(1000, 14)

HT_IZ <- c(height0,height8,height16,height24,height32)

Heights<-rep(seq(1,1000),5)

Groups <- c(rep("height0",1000), rep("height8",1000), rep("height16",1000), rep("height24",1000), rep("height32",1000))

AllData <- data.frame(HT_IZ,Heights,Groups)

g13 <- ggboxplot(AllData,
                x = "Groups",
                y = "HT_IZ", 
                color = "Groups",
                fill = "Groups", palette = c("#DCE319FF", "#73D055FF", "#238A8DFF", "#39568CFF", "#440154FF"), alpha=0.8,
                order = c("height0", "height8", "height16", "height24", "height32"),
                ylab = "Tipulidae s.l. richness",
                xlab = "Height categories")

g13 + coord_flip()
```

---

# Drosophilidae richness

```
height0  <- rpois(1000, 18)
height8  <- rpois(1000, 16)
height16 <- rpois(1000, 14)
height24 <- rpois(1000, 19)
height32 <- rpois(1000, 6)

HT_IZ <- c(height0,height8,height16,height24,height32)

Heights<-rep(seq(1,1000),5)

Groups <- c(rep("height0",1000), rep("height8",1000), rep("height16",1000), rep("height24",1000), rep("height32",1000))

AllData <- data.frame(HT_IZ,Heights,Groups)


g14 <- ggboxplot(AllData,
                x = "Groups",
                y = "HT_IZ", 
                color = "Groups",
                fill = "Groups", palette = c("#DCE319FF", "#73D055FF", "#238A8DFF", "#39568CFF", "#440154FF"), alpha=0.8,
                order = c("height0", "height8", "height16", "height24", "height32"),
                ylab = "Drosophilidae richness",
                xlab = "Height categories")

g14 + coord_flip()
```

---

# Lauxaniidae richness

```
height0  <- rpois(1000, 14)
height8  <- rpois(1000, 19)
height16 <- rpois(1000, 22)
height24 <- rpois(1000, 21)
height32 <- rpois(1000, 5)

HT_IZ <- c(height0,height8,height16,height24,height32)

Heights<-rep(seq(1,1000),5)

Groups <- c(rep("height0",1000), rep("height8",1000), rep("height16",1000), rep("height24",1000), rep("height32",1000))

AllData <- data.frame(HT_IZ,Heights,Groups)


g15 <- ggboxplot(AllData,
                x = "Groups",
                y = "HT_IZ", 
                color = "Groups",
                fill = "Groups", palette = c("#DCE319FF", "#73D055FF", "#238A8DFF", "#39568CFF", "#440154FF"), alpha=0.8,
                order = c("height0", "height8", "height16", "height24", "height32"),
                ylab = "Lauxaniidae richness",
                xlab = "Height categories")

g15 + coord_flip()
```

---

# Phoridae genus richness

```
height0  <- rpois(1000, 19)
height8  <- rpois(1000, 27)
height16 <- rpois(1000, 19)
height24 <- rpois(1000, 22)
height32 <- rpois(1000, 16)

HT_IZ <- c(height0,height8,height16,height24,height32)

Heights<-rep(seq(1,1000),5)

Groups <- c(rep("height0",1000), rep("height8",1000), rep("height16",1000), rep("height24",1000), rep("height32",1000))

AllData <- data.frame(HT_IZ,Heights,Groups)


g16 <- ggboxplot(AllData,
                x = "Groups",
                y = "HT_IZ", 
                color = "Groups",
                fill = "Groups", palette = c("#DCE319FF", "#73D055FF", "#238A8DFF", "#39568CFF", "#440154FF"), alpha=0.8,
                order = c("height0", "height8", "height16", "height24", "height32"),
                ylab = "Phoridae genus richness",
                xlab = "Height categories")

g16 + coord_flip()
```
