# Supplementary material for: Vertical stratification of insect abundance and species richness in an Amazonian tropical forest
Source: Sci Rep. 2022 Feb 2;12:1734. doi: 10.1038/s41598-022-05677-y (PMC8810858; doi:10.1038/s41598-022-05677-y)
Supplement: Supplementary file 2 — Supplementary Table S1. [file 41598_2022_5677_MOESM2_ESM.pdf]

Vertical stratification of insect abundance and species richness in an Amazonian tropical forest

Amorim et al.

Scientific Reports

Supplementary Material Table S1. Abundance of insect orders sampled at each level of the ZF2 biological reserve tower. Patterns were built only for families with 10 or more specimens.

	Total number of specimens						% of order abundance at level							
Insect orders	0 m	8 m	16 m	24 m	32 m	TOTAL	0 m	8 m	16 m	24 m	32 m	8m-32m abundance	Peaks	Peak levels
Diptera	5767	3238	1947	3586	2062	16600	34,70%	19,50%	11,70%	21,60%	12,40%	65,30%	2p	2p(0/16+24)
Hymenoptera	3331	1230	950	1157	611	7279	45,80%	16,90%	13,10%	15,90%	8,40%	54,20%	2p	2p(0/16+24)
Lepidoptera	776	1049	1281	2820	973	6899	11,20%	15,20%	18,60%	40,90%	14,10%	88,80%	1p	1p(16+24)
Hemiptera	631	1033	834	1049	392	3939	16,00%	26,20%	21,20%	26,60%	10,00%	84,00%	2p	2p(8/24)
Coleoptera	1054	607	392	463	154	2670	39,50%	22,70%	14,70%	17,30%	5,80%	60,50%	2p	2p(0/16+24)
Blattaria	37	23	60	72	38	230	16,10%	10,00%	26,10%	31,30%	16,50%	83,90%	1p	1p(16+24)
Psocoptera	93	40	21	7		161	57,80%	24,80%	13,00%	4,30%	0,00%	42,20%	1p	1p(0)
Orthoptera	33	29	22	29	15	128	25,80%	22,70%	17,20%	22,70%	11,70%	74,20%	2p	2p(0/16+24)
Trichoptera	11	6		9	45	71	15,50%	8,50%	0,00%	12,70%	63,40%	84,50%	1p	1p(32)
Neuroptera	7	17	11	18	7	60	11,70%	28,30%	18,30%	30,00%	11,70%	88,30%	2p	2p(8/16+24)
Collembola	44					44	100%	0,00%	0,00%	0,00%	0,00%	0,00%	1p	1p(0)
Isoptera	17	13	3			33	51,50%	39,40%	9,10%	0,00%	0,00%	48,50%	1p	1p(0)
Thysanoptera	2	1	2	2		7	28,60%	14,30%	28,60%	28,60%	0,00%	71,40%		
Mantodea		1	2	2		5	0,00%	20,00%	40,00%	40,00%	0,00%	100%		
Dermaptera				2		2	0,00%	0,00%	0,00%	100%	0,00%	100%		
Archaeognatha			1			1	0,00%	0,00%	100%	0,00%	0,00%	100%		
Odonata			1			1	0,00%	0,00%	100%	0,00%	0,00%	100%		
Phasmatodea			1			1	0,00%	0,00%	100%	0,00%	0,00%	100%		
TOTAL	11689	7220	5485	9154	4230	37778	30,90%	19,10%	14,50%	24,20%	11,20%	69,10%		
