# Supplementary material for: Vertical stratification of insect abundance and species richness in an Amazonian tropical forest
Source: Sci Rep. 2022 Feb 2;12:1734. doi: 10.1038/s41598-022-05677-y (PMC8810858; doi:10.1038/s41598-022-05677-y)
Supplement: Supplementary file 3 — Supplementary Table S2. [file 41598_2022_5677_MOESM3_ESM.pdf]

Vertical stratification of insect abundance and species richness in an Amazonian tropical forest
Amorim et al.
Scientific Reports

Supplementary Material Table S2. Abundance (total number of specimens) of each fly family sampled at each level of the ZF2 biological reserve tower. Patterns were built only for families with 10 or more specimens.

	Number of specimens						Peaks / peak-pattern		% of specimens				
Family	0 m	8 m	16 m	24 m	32 m	TOTAL	Peaks	Pattern	0 m	8 m	16 m	24 m	32 m
Phoridae	1950	656	206	723	256	3843	2	2p(0/16+24)	50,7%	17,1%	5,4%	18,8%	6,7%
Sciaridae	423	571	282	486	304	2066	2	2p(8/24)	20,5%	27,6%	13,6%	23,5%	14,7%
Cecidomyiidae	431	456	362	391	337	1977	-	no peak	21,8%	23,1%	18,3%	19,8%	17,0%
Psychodidae	472	318	79	298	11	1178	2	2p(0/16+24)	40,1%	27,0%	6,7%	25,3%	0,9%
Dolichopodidae	183	75	192	319	166	935	2	2p(0/16+24)	19,6%	8,0%	20,5%	34,1%	17,8%
Chironomidae	342	130	112	141	155	880	1	2p-0/32	38,9%	14,8%	12,7%	16,0%	17,6%
Tachinidae	49	220	158	140	266	833	2	2p(8/32)	5,9%	26,4%	19,0%	16,8%	31,9%
Mycetophilidae	554	78	46	61	58	797	1	1p(0)	69,5%	9,8%	5,8%	7,7%	7,3%
Tipulidae s.l.	321	78	59	167	113	738	2	2p(0/16+24)	43,5%	10,6%	8,0%	22,6%	15,3%
Ceratopogonidae	142	91	59	195	142	629	2	2p(0/16+24)	22,6%	14,5%	9,4%	31,0%	22,6%
Milichiidae	207	126	36	56	10	435	1	1p(0)	47,6%	29,0%	8,3%	12,9%	2,3%
Lonchaeidae	21	43	84	173	112	433	1	1p(16+24)	4,8%	9,9%	19,4%	40,0%	25,9%
Lauxaniidae	21	54	73	118	16	282	1	1p(16+24)	7,4%	19,1%	25,9%	41,8%	5,7%
Culicidae	93	46	28	43	2	212	2	2p(0/16+24)	43,9%	21,7%	13,2%	20,3%	0,9%
Empididae	57	30	29	79	9	211	2	2p(0/16+24)	27,0%	14,2%	13,7%	37,4%	4,3%
Keroplatidae	71	53	10	6	0	140	1	1p(0)	50,7%	37,9%	7,1%	4,3%	0,0%
Muscidae	39	22	10	34	31	136	2	2p(0/16+24)	28,7%	16,2%	7,4%	25,0%	22,8%
Drosophilidae	32	21	18	34	12	117	2	2p(0/16+24)	27,4%	17,9%	15,4%	29,1%	10,3%
Asilidae	80	10	9	10	7	116	1	1p(0)	69,0%	8,6%	7,8%	8,6%	6,0%
Tabanidae	58	19	9	5	4	95	1	1p(0)	61,1%	20,0%	9,5%	5,3%	4,2%
Sphaeroceridae	59	6	6	9	0	80	1	1p(0)	73,8%	7,5%	7,5%	11,3%	0,0%
Chloropidae	22	27	7	22	1	79	2	2p(8/24)	27,8%	34,2%	8,9%	27,8%	1,3%
Stratiomyidae	49	15	11	2	1	78	1	1p(0)	62,8%	19,2%	14,1%	2,6%	1,3%
Sarcophagidae	4	1	1	3	30	39	1	1p(32)	10,3%	2,6%	2,6%	7,7%	76,9%
Corethrelidae	17	10	0	4	0	31	2	2p(0/16+24)	54,8%	32,3%	0,0%	12,9%	0,0%
Ephydridae	9	6	4	9	0	28	2	2p(0/16+24)	32,1%	21,4%	14,3%	32,1%	0,0%
Syrphidae	0	6	9	8	1	24	1	1p(16+24)	0,0%	25,0%	37,5%	33,3%	4,2%
Clusiidae	2	9		8	1	20	2	2p(8/24)	10,0%	45,0%	0,0%	40,0%	5,0%
Odiniidae	2	2	5	9	2	20	1	1p(16+24)	10,0%	10,0%	25,0%	45,0%	10,0%
Periscelididae	6	13	1	0	0	20	1	1p(8)	30,0%	65,0%	5,0%	0,0%	0,0%
Micropezidae	7	6	3	3	0	19	1	1p(0)	36,8%	31,6%	15,8%	15,8%	0,0%
Agromyzidae	2	2	6	0	7	17	2	2p(16+24/32)	11,8%	11,8%	35,3%	0,0%	41,2%
Anisopodidae	0	7	6	3	0	16	1	1p(8)	0,0%	43,8%	37,5%	18,8%	0,0%
Bibionidae	9	0	2		0	11	2	2p(0/16+24)	81,8%	0,0%	18,2%	0,0%	0,0%
Lygistorrhinidae	6	8	1	0	0	15	1	1p(0)	40,0%	53,3%	6,7%	0,0%	0,0%
Pipunculidae	2	4	3	6	0	15	1	1p(16+24)	13,3%	26,7%	20,0%	40,0%	0,0%
Calliphoridae	0	4	6	4	1	15	1	1p(16+24)	0,0%	26,7%	40,0%	26,7%	6,7%
Scatopsidae	5	6	1	1	0	13	1	1p(8)	38,5%	46,2%	7,7%	7,7%	0,0%
Tephritidae	0	0	7	5	0	12	1	1p(16+24)	0,0%	0,0%	58,3%	41,7%	0,0%
Ulidiidae	4	2	0	1	3	10	2	2p-0/32	40,0%	20,0%	0,0%	10,0%	30,0%
Richardiidae	0	0	4	0	3	7			0,0%	0,0%	57,1%	0,0%	42,9%
Conopidae	2	2	0	2	0	6			33,3%	33,3%	0,0%	33,3%	0,0%
Sepsidae	5	1	0	0	0	6			83,3%	16,7%	0,0%	0,0%	0,0%
Therevidae	0	0	0	5	0	5			0,0%	0,0%	0,0%	100,0%	0,0%
Neriidae	0	1	3	0	0	4			0,0%	25,0%	75,0%	0,0%	0,0%
Rhagionidae	2	0	0	1	0	3			66,7%	0,0%	0,0%	33,3%	0,0%
Bombyliidae	0	1	0	1	0	2			0,0%	50,0%	0,0%	50,0%	0,0%
Inbiomyiidae	2	0	0	0	0	2			100%	0,0%	0,0%	0,0%	0,0%
Rhinophoridae	2	0	0	0	0	2			100%	0,0%	0,0%	0,0%	0,0%
Diadocidiidae	1	0	0	0	0	1			100%	0,0%	0,0%	0,0%	0,0%
Ditomyiidae	1	0	0	0	0	1			100%	0,0%	0,0%	0,0%	0,0%
Platypezidae	0	0	0	1	0	1			0,0%	0,0%	0,0%	100%	0,0%
Aulacigastridae	1	0	0	0	0	1			100%	0,0%	0,0%	0,0%	0,0%
Heleomyzidae	0	1	0	0	0	1			0,0%	100%	0,0%	0,0%	0,0%
Pseudopomyzidae	0	0	0	0	1	1			0,0%	0,0%	0,0%	0,0%	100%
Anthomyiidae	0	1	0	0	0	1			0,0%	100%	0,0%	0,0%	0,0%
TOTAL	5767	3238	1947	3586	2062	16600			34,7%	19,5%	11,7%	21,6%	12,4%
