# Supplementary material for: Vertical stratification of insect abundance and species richness in an Amazonian tropical forest
Source: Sci Rep. 2022 Feb 2;12:1734. doi: 10.1038/s41598-022-05677-y (PMC8810858; doi:10.1038/s41598-022-05677-y)
Supplement: Supplementary file 4 — Supplementary Table S3. [file 41598_2022_5677_MOESM4_ESM.pdf]

Vertical stratification of insect abundance and species richness in an Amazonian tropical forest

Amorim et al.

Scientific Reports

Supplementary Material Table S3. Number of species of fly families sampled at each level of the ZF2 biological reserve tower. Patterns only for families with more than five specimens.

	Number of species						Peaks / peak-pattern		% at each level				
Diptera family	0 m	8 m	16 m	24 m	32 m	TOTAL	Peaks	Pattern	0 m	8 m	16 m	24 m	32 m
Tachinidae	17	73	66	32	61	166	2	2p(8/32)	10,2%	44,0%	39,8%	19,3%	36,7%
Mycetophilidae*	79	25	20	20	9	101	1	1p(0)	78,2%	24,8%	19,8%	19,8%	8,9%
Dolichopodidae	30	22	42	34	28	71	2	2p(0/16+24)	42,3%	31,0%	59,2%	47,9%	39,4%
Tipulidae s.l.	43	22	15	28	14	78	2	2p(0/16+24)	55,1%	28,2%	19,2%	35,9%	17,9%
Drosophilidae	18	16	14	19	6	51	2	2p(0/16+24)	35,3%	31,4%	27,5%	37,3%	11,8%
Phoridae**	19	27	19	22	16	46	2	2p(8/24)	41,3%	58,7%	41,3%	47,8%	34,8%
Lauxaniidae	14	19	22	21	5	46	1	1p(16+24)	30,4%	41,3%	47,8%	45,7%	10,9%
Muscidae	8	9	9	14	13	41	1	1p(16+24)	19,5%	22,0%	22,0%	34,1%	31,7%
Milichiidae	17	7	9	17	4	37	2	2p(0/16+24)	45,9%	18,9%	24,3%	45,9%	10,8%
Stratiomyidae	19	7	9	2	1	32	2	2p(0/16+24)	59,4%	21,9%	28,1%	6,3%	3,1%
Chloropidae	9	13	4	7	1	29	2	2p(8/24)	31,0%	44,8%	13,8%	24,1%	3,4%
Empididae	15	11	6	7	3	24	2	2p(0/16+24)	62,5%	45,8%	25,0%	29,2%	12,5%
Tabanidae	12	10	4	5	4	19	2	2p(0/16+24)	63,2%	52,6%	21,1%	26,3%	21,1%
Clusiidae	1	7	0	7	1	16	2	2p(8/24)	6,3%	43,8%	0,0%	43,8%	6,3%
Pipunculidae	2	4	3	6	0	14	2	2p(8/24)	14,3%	28,6%	21,4%	42,9%	0,0%
Syrphidae	0	5	6	6	1	14	1	1p(16+24)	0,0%	35,7%	42,9%	42,9%	7,1%
Sarcophagidae	2	1	1	3	6	12	2	2p(0/32)	16,7%	8,3%	8,3%	25,0%	50,0%
Micropezidae	6	3	3	3	0	10	1	1p(0)	60,0%	30,0%	30,0%	30,0%	0,0%
Odiniidae	1	2	3	6	2	8	1	1p(16+24)	12,5%	25,0%	37,5%	75,0%	25,0%
Scatopsidae	4	5	1	1	0	7	1	1p(8)	57,1%	71,4%	14,3%	14,3%	0,0%
Anisopodidae	0	5	3	1	0	5			0,0%	100%	60,0%	20,0%	0,0%
Lygistorrhinidae	1	3	1	0	0	4			25,0%	75,0%	25,0%	0,0%	0,0%
Conopidae	1	1	0	1	0	3			33,3%	33,3%	0,0%	33,3%	0,0%
Rhagionidae	2	0	0	1	0	3			66,7%	0,0%	0,0%	33,3%	0,0%
Sepsidae	2	1	0	0	0	3			66,7%	33,3%	0,0%	0,0%	0,0%
Rhinophoridae	2	0	0	0	0	2			100%	0,0%	0,0%	0,0%	0,0%
Neriidae	0	1	2	0	0	2			0,0%	50,0%	100%	0,0%	0,0%
Bibionidae	1	0	1	0	0	2			50,0%	0,0%	50,0%	0,0%	0,0%
Platypezidae	0	0	0	1	0	1			0,0%	0,0%	0,0%	100%	0,0%
Anthomyiidae	0	1	0	0	0	1			0,0%	100%	0,0%	0,0%	0,0%
Heleomyzidae	0	1	0	0	0	1			0,0%	100%	0,0%	0,0%	0,0%
Inbiomyiidae	1	0	0	0	0	1			100%	0,0%	0,0%	0,0%	0,0%
Pseudopomyzidae	0	0	0	0	1	1			0,0%	0,0%	0,0%	0,0%	100%
Aulacigastridae	1	0	0	0	0	1			100%	0,0%	0,0%	0,0%	0,0%
Bombyliidae	0	1	0	1	0	1			0,0%	100%	0,0%	100%	0,0%
Therevidae	0	0	0	1	0	1			0,0%	0,0%	0,0%	100%	0,0%
Diadocidiidae	1	0	0	0	0	1			100%	0,0%	0,0%	0,0%	0,0%
Ditomyiidae	1	0	0	0	0	1			100%	0,0%	0,0%	0,0%	0,0%
TOTAL	312	229	197	234	115	856			36,4%	26,8%	23,0%	27,3%	13,4%

* The mycetophilid genus Manota identified only to genus and counted as one species

** Identified to genera, each genus counted as one species
