# Supplementary material for: Vertical stratification of insect abundance and species richness in an Amazonian tropical forest
Source: Sci Rep. 2022 Feb 2;12:1734. doi: 10.1038/s41598-022-05677-y (PMC8810858; doi:10.1038/s41598-022-05677-y)
Supplement: Supplementary file 5 — Supplementary Table S4. [file 41598_2022_5677_MOESM5_ESM.pdf]

Vertical stratification of insect abundance and species richness in an Amazonian tropical forest

Amorim et al.

Scientific Reports

Supplementary Material Table S4. Number of species of different Diptera guilds at each level of the ZF2 biological reserve tower.

	N° of spp	%	0 m	8 m	16 m	24 m	32 m
Unknown biology	116	-	-	-	-	-	-
Parasitoids	207	28,1%	31	87	77	53	53
Phytosaprophagous	162	22,0%	70	52	46	53	14
Predators	157	21,3%	65	54	62	67	43
Fungivorous	114	15,5%	83	30	22	23	12
Scavengers	34	4,6%	11	8	9	17	10
Kleptoparasites	16	2,2%	7	7	7	7	7
Ant and termite nest associates	14	1,9%	4	1	3	8	1
Sap flux	13	1,8%	5	6	6	2	0
Herbivores	10	1,4%	5	1	1	0	0
Coprophagous	9	1,2%	5	2	1	1	2
