# Supplementary material for: Vertical stratification of insect abundance and species richness in an Amazonian tropical forest
Source: Sci Rep. 2022 Feb 2;12:1734. doi: 10.1038/s41598-022-05677-y (PMC8810858; doi:10.1038/s41598-022-05677-y)
Supplement: Supplementary file 6 — Supplementary Table S5. [file 41598_2022_5677_MOESM6_ESM.pdf]

Vertical stratification of insect abundance and species richness in an Amazonian tropical forest
Amorim et al.
Scientific Reports
Supplementary Material Table S5. Full data on identified species of Diptera in the ZF2 biological reserve tower samples.

			Total abundance				
**Family**	**Subfamily**	**Species**	0m	8 m	16 m	24 m	32 m
Tipulidae s.l.	Chioneinae	*Atarba* sp. 1	0	0	0	1	1
Tipulidae s.l.	Chioneinae	*Atarba* sp. 2	0	0	0	1	0
Tipulidae s.l.	Chioneinae	*Atarba* sp. 3	7	0	0	0	0
Tipulidae s.l.	Chioneinae	*Atarba* sp. 4	1	0	2	0	0
Tipulidae s.l.	Chioneinae	*Atarba* sp. 5	0	0	0	1	0
Tipulidae s.l.	Chioneinae	*Atarba* sp. 6	0	0	0	2	0
Tipulidae s.l.	Chioneinae	*Erioptera (Mesocyphona)* sp. 1	19	10	0	9	0
Tipulidae s.l.	Chioneinae	*Gnophomyia* sp. 1	18	1	0	5	3
Tipulidae s.l.	Chioneinae	*Gnophomyia* sp. 2	1	0	0	0	0
Tipulidae s.l.	Chioneinae	*Gnophomyia* sp. 3	3	0	0	0	0
Tipulidae s.l.	Chioneinae	*Gonomyia* sp. 1	0	1	0	1	0
Tipulidae s.l.	Chioneinae	*Styringomyia* sp. 1	1	0	0	0	0
Tipulidae s.l.	Chioneinae	*Teucholabis* (*T.*) sp. 1	27	0	0	3	0
Tipulidae s.l.	Chioneinae	*Teucholabis* (*T.*) sp. 2	9	2	0	0	0
Tipulidae s.l.	Chioneinae	*Teucholabis* (*T.*) sp. 3	1	1	1	5	1
Tipulidae s.l.	Chioneinae	*Teucholabis* (*T.*) sp. 4	5	2	0	1	0
Tipulidae s.l.	Chioneinae	*Teucholabis* (*T.*) sp. 5	0	2	0	10	3
Tipulidae s.l.	Chioneinae	*Teucholabis* (*T.*) sp. 6	0	0	0	1	0
Tipulidae s.l.	Chioneinae	*Teucholabis* (*T.*) sp. 7	1	0	0	0	0
Tipulidae s.l.	Chioneinae	*Teucholabis* (*T.*) sp. 8	1	0	2	9	0
Tipulidae s.l.	Chioneinae	*Teucholabis* (*T.*) sp. 9	0	0	0	9	1
Tipulidae s.l.	Chioneinae	*Teucholabis* (*T.*) sp. 10	2	0	0	1	0
Tipulidae s.l.	Chioneinae	*Teucholabis* (*T.*) sp. 11	2	0	0	0	0
Tipulidae s.l.	Chioneinae	*Teucholabis* (*T.*) sp. 12	1	0	0	0	0
Tipulidae s.l.	Chioneinae	*Teucholabis* (*T.*) sp. 13	47	0	0	0	0
Tipulidae s.l.	Chioneinae	*Teucholabis* (*T.*) sp. 14	0	0	0	1	0
Tipulidae s.l.	Chioneinae	*Teucholabis* (*T.*) sp. 15	1	0	0	0	0
Tipulidae s.l.	Chioneinae	*Teucholabis* (*T.*) sp. 16	33	0	1	0	0
Tipulidae s.l.	Chioneinae	*Teucholabis* (*T.*) sp. 17	0	0	1	0	0
Tipulidae s.l.	Chioneinae	*Teucholabis* (*T.*) sp. 18	23	0	0	0	0
Tipulidae s.l.	Limnophilinae	*Hexatoma* sp. 1	1	1	0	0	0
Tipulidae s.l.	Limnophilinae	*Polymera* sp. 1	0	2	0	0	0
Tipulidae s.l.	Limnophilinae	*Polymera* sp. 2	0	0	0	1	0
Tipulidae s.l.	Limnophilinae	*Polymera* sp. 3	1	0	0	0	0
Tipulidae s.l.	Limoniinae	*Dicranomyia* sp. 1	2	0	0	0	0
Tipulidae s.l.	Limoniinae	*Dicranomyia* sp. 2	2	0	0	0	0
Tipulidae s.l.	Limoniinae	*Dicranomyia* sp. 3	3	0	0	0	0
Tipulidae s.l.	Limoniinae	*Dicranomyia* sp. 4	2	0	0	0	0
Tipulidae s.l.	Limoniinae	*Dicranomyia* sp. 5	13	0	0	0	0
Tipulidae s.l.	Limoniinae	*Dicranomyia* sp. 6	0	1	0	0	0
Tipulidae s.l.	Limoniinae	*Dicranomyia* sp. 7	0	1	0	0	0
Tipulidae s.l.	Limoniinae	*Dicranomyia* sp. 8	0	22	38	81	92
Tipulidae s.l.	Limoniinae	*Dicranomyia* sp. 9	0	0	0	1	0
Tipulidae s.l.	Limoniinae	*Elephantomyia* sp. 1	1	0	0	0	0
Tipulidae s.l.	Limoniinae	*Geranomyia* sp. 1	2	2	1	0	0
Tipulidae s.l.	Limoniinae	*Geranomyia* sp. 2	0	0	1	0	0
Tipulidae s.l.	Limoniinae	*Geranomyia* sp. 3	0	0	0	0	1
Tipulidae s.l.	Limoniinae	*Helius* sp. 1	3	0	6	7	0
Tipulidae s.l.	Limoniinae	*Helius* sp. 2	2	5	2	0	0
Tipulidae s.l.	Limoniinae	*Helius* sp. 3	3	0	0	0	0
Tipulidae s.l.	Limoniinae	*Helius* sp. 4	0	1	0	0	0

Tipulidae s.l.	Limoniinae	*Rhipidia* sp. 1	0	0	0	0	1
Tipulidae s.l.	Limoniinae	*Toxorhina* sp. 1	1	0	0	0	0
Tipulidae s.l.	Limoniinae	*Toxorhina* sp. 2	0	0	2	1	0
Tipulidae s.l.	Limoniinae	*Toxorhina* sp. 3	0	0	0	1	0
Tipulidae s.l.	Limoniinae	*Toxorhina* sp. 4	0	5	0	0	0
Tipulidae s.l.	Limoniinae	*Toxorhina* sp. 5	0	2	0	0	0
Tipulidae s.l.	-	INDET sp. 1	0	0	1	0	0
Tipulidae s.l.	-	INDET sp. 2	0	0	0	1	0
Tipulidae s.l.	-	INDET sp. 3	0	0	0	0	2
Tipulidae s.l.	Tipulinae	*Brachypremna* sp. 1	2	1	1	1	0
Tipulidae s.l.	Tipulinae	*Leptotarsus* sp. 1	2	0	0	0	0
Tipulidae s.l.	Tipulinae	*Leptotarsus* sp. 2	46	8	0	0	0
Tipulidae s.l.	Tipulinae	*Leptotarsus* sp. 3	1	0	0	0	0
Tipulidae s.l.	Tipulinae	*Leptotarsus* sp. 4	1	0	0	0	0
Tipulidae s.l.	Tipulinae	*Leptotarsus* sp. 5	0	0	0	0	1
Tipulidae s.l.	Tipulinae	*Tipula* sp. 1	0	1	0	0	0
Tipulidae s.l.	Tipulinae	*Tipula* sp. 2	0	0	0	1	0
Tipulidae s.l.	Tipulinae	*Tipula* (*Eumicrotipula*) sp. 1	1	1	1	5	2
Tipulidae s.l.	Tipulinae	*Tipula* (*Microtipula*) sp. 1	25	0	0	0	0
Tipulidae s.l.	Tipulinae	*Tipula* (*Microtipula*) sp. 2	0	0	0	0	1
Tipulidae s.l.	Tipulinae	*Tipula* (*Microtipula*) sp. 3	0	0	6	0	0
Tipulidae s.l.	Tipulinae	*Tipula* (*Microtipula*) sp. 4	0	0	0	0	2
Tipulidae s.l.	Tipulinae	*Tipula* (*Microtipula*) sp. 5	0	6	0	6	2
Tipulidae s.l.	Tipulinae	*Tipula* (*Microtipula*) sp. 6	1	0	0	0	0
Tipulidae s.l.	Tipulinae	*Tipula* (*Microtipula*) sp. 7	0	0	0	1	0
Tipulidae s.l.	Tipulinae	*Tipula* (*Microtipula*) sp. 8	1	0	0	0	0
Tipulidae s.l.	Tipulinae	*Zelandotipula* sp. 1	2	0	0	0	0
Anisopodidae	Olbiogastrinae	*Olbiogaster* sp. 1	0	2	2	0	0
Anisopodidae	Olbiogastrinae	*Olbiogaster* sp. 2	0	2	2	3	0
Anisopodidae	Olbiogastrinae	*Olbiogaster* sp. 3	0	1	2	0	0
Anisopodidae	Olbiogastrinae	*Olbiogaster* sp. 4	0	1	0	0	0
Anisopodidae	Olbiogastrinae	*Olbiogaster* sp. 5	0	1	0	0	0
Bibionidae	Pleciinae	*Plecia* sp. 1	9	0	0	0	0
Bibionidae	Pleciinae	Plecia *sp. 2*	0	0	2	0	0
Diadocidiidae		*Diadocidia* sp. 1	1	0	0	0	0
Ditomyiidae		*Melosymmerus* sp. 1	1	0	0	0	0
Lygistorrhinidae		*Lygistorrhina* sp. 1	0	2	0	0	0
Lygistorrhinidae		*Lygistorrhina* sp. 2	6	2	0	0	0
Lygistorrhinidae		*Lygistorrhina* sp. 3	0	4	0	0	0
Lygistorrhinidae		*Lygistorrhina* sp. 4	0	0	1	0	0
Mycetophilidae	Sciophilinae	*Cluzobra* sp. 1	0	0	0	1	0
Mycetophilidae	Sciophilinae	*Cluzobra* sp. 2	26	19	8	1	0
Mycetophilidae	Sciophilinae	*Cluzobra* sp. 3	0	2	1	0	0
Mycetophilidae	Sciophilinae	*Cluzobra* sp. 4	1	1	1	0	0
Mycetophilidae	Sciophilinae	*Cluzobra variegata* Matile	0	2	1	0	0
Mycetophilidae	Sciophilinae	*Cluzobra* sp. 6	0	0	1	0	0
Mycetophilidae	Sciophilinae	*Cluzobra* sp. 7	22	1	0	0	0
Mycetophilidae	Sciophilinae	*Cluzobra* sp. 8	20	0	0	0	0
Mycetophilidae	Sciophilinae	*Cluzobra unident. Females*	12	7	9	0	0
Mycetophilidae	Sciophilinae	*Monoclona* sp. 1	1	1	0	0	0
Mycetophilidae	Sciophilinae	*Monoclona* sp. 2	0	1	0	0	0
Mycetophilidae	Sciophilinae	*Monoclona* sp. 3	2	0	0	0	0
Mycetophilidae	Gnoristinae	*Dziedzickia* sp. 1	11	0	0	0	0
Mycetophilidae	Gnoristinae	*Dziedzickia* sp. 2	3	0	0	0	0
Mycetophilidae	Gnoristinae	*Dziedzickia* sp. 3	1	0	0	0	0
Mycetophilidae	Gnoristinae	*Dziedzickia* sp. 4	1	0	0	0	0
Mycetophilidae	Gnoristinae	*Dziedzickia* sp. 5	1	0	0	0	0
Mycetophilidae	Gnoristinae	*Schnusea* sp. 1	1	0	0	0	0
Mycetophilidae	Gnoristinae	*Schnusea* sp. 2	2	0	0	0	0
Mycetophilidae	Gnoristinae	*Schnusea* sp. 3	18	0	0	0	0
Mycetophilidae	Gnoristinae	*Synapha* sp. 1	6	2	1	0	7
Mycetophilidae	Gnoristinae	*Synapha* sp. 2	0	0	0	0	7
Mycetophilidae	Gnoristinae	*Synapha* sp. 3	1	0	0	0	0

Mycetophilidae	Gnoristinae	*Synapha* sp. 4	1	0	0	0	0
Mycetophilidae	Gnoristinae	*Synapha* sp. 5	1	0	0	0	0
Mycetophilidae	Gnoristinae	Genus E sp. 1	1	0	0	0	0
Mycetophilidae	Leiinae	*Aphrastomyia cerquerai* Lane	1	0	1	0	0
Mycetophilidae	Leiinae	*Aphrastomyia* sp. 2	3	0	0	0	0
Mycetophilidae	Leiinae	*Aphrastomyia* sp. 3	2	0	0	0	0
Mycetophilidae	Leiinae	*Leia* sp. 1	28	7	3	8	24
Mycetophilidae	Leiinae	*Leia* sp. 2	15	1	4	14	11
Mycetophilidae	Leiinae	*Leia* sp. 3	22	0	1	15	3
Mycetophilidae	Leiinae	*Leia* sp. 4	0	0	0	1	0
Mycetophilidae	Leiinae	*Leia* sp. 5	0	0	1	0	0
Mycetophilidae	Leiinae	*Leiella unicincta* Edwards	0	0	0	1	1
Mycetophilidae	Leiinae	*Leiella zonalis* Edwards	0	3	0	5	0
Mycetophilidae	Leiinae	*Leiella* sp. 3	1	0	0	0	0
Mycetophilidae	Leiinae	*Leiella* sp. 4	2	0	0	0	0
Mycetophilidae	Leiinae	*Manota* spp.	155	9	1	0	1
Mycetophilidae	Tetragoneurinae	*Tetragoneura* sp. 1	2	3	0	0	0
Mycetophilidae	Tetragoneurinae	*Tetragoneura* sp. 2	2	4	0	0	0
Mycetophilidae	Tetragoneurinae	*Tetragoneura* sp. 3	2	1	0	0	0
Mycetophilidae	Tetragoneurinae	*Tetragoneura* sp. 4	5	1	0	0	0
Mycetophilidae	Mycomyinae	*Mycomya* sp. 1	0	1	1	1	1
Mycetophilidae	Mycomyinae	*Mycomya* sp. 2	0	1	0	0	0
Mycetophilidae	Mycomyinae	*Neoempheria* sp. 1	0	0	2	1	0
Mycetophilidae	Mycomyinae	*Neoempheria* sp. 2	0	0	0	1	0
Mycetophilidae	Mycomyinae	*Neoempheria* sp. 3	0	0	0	3	0
Mycetophilidae	Mycomyinae	*Neoempheria* sp. 4	1	0	0	1	0
Mycetophilidae	Mycomyinae	*Neoempheria* sp. 5	1	0	0	0	0
Mycetophilidae	Mycomyinae	*Neoempheria* sp. 6	1	0	0	0	0
Mycetophilidae	Mycomyinae	*Neoempheria* sp. 7	1	0	0	0	0
Mycetophilidae	Mycomyinae	*Neoempheria* sp. 8	2	0	0	0	0
Mycetophilidae	Mycomyinae	*Neoempheria* sp. 9	1	0	0	0	0
Mycetophilidae	Mycomyinae	*Neoempheria* sp. 10	1	0	0	0	0
Mycetophilidae	Mycomyinae	*Neoempheria* sp. 11	1	0	0	0	0
Mycetophilidae	Mycomyinae	*Neoempheria* sp. 12	1	0	0	0	0
Mycetophilidae	Mycomyinae	*Neoempheria* sp. 13	1	0	0	0	0
Mycetophilidae	Mycomyinae	*Neoempheria* sp. 14	1	0	0	0	0
Mycetophilidae	Mycomyinae	*Neoempheria* sp. 15	1	0	0	0	0
Mycetophilidae	Mycomyinae	*Neoempheria* sp. 16	3	0	0	0	0
Mycetophilidae	Mycomyinae	*Neoempheria* sp. 17	1	0	0	0	0
Mycetophilidae	Mycomyinae	*Neoempheria* sp. 18	2	0	0	0	0
Mycetophilidae	Mycomyinae	*Neoempheria* sp. 19	1	0	0	0	0
Mycetophilidae	Mycomyinae	*Neoempheria* sp. 20	1	0	0	0	0
Mycetophilidae	Mycomyinae	*Neoempheria* sp. 21	1	0	0	0	0
Mycetophilidae	Mycetophilinae	*Epicypta* sp. 1	4	1	0	1	2
Mycetophilidae	Mycetophilinae	*Epicypta* sp. 2	1	0	0	1	0
Mycetophilidae	Mycetophilinae	*Epicypta* sp. 3	0	0	0	1	0
Mycetophilidae	Mycetophilinae	*Epicypta* sp. 4	0	0	0	1	0
Mycetophilidae	Mycetophilinae	*Epicypta* sp. 5	2	0	0	1	0
Mycetophilidae	Mycetophilinae	*Epicypta* sp. 6	30	1	2	2	0
Mycetophilidae	Mycetophilinae	*Epicypta* sp. 7	4	0	0	1	0
Mycetophilidae	Mycetophilinae	*Epicypta* sp. 8	2	2	0	0	1
Mycetophilidae	Mycetophilinae	*Epicypta* sp. 9	2	0	1	0	0
Mycetophilidae	Mycetophilinae	*Epicypta* sp. 10	4	0	2	0	0
Mycetophilidae	Mycetophilinae	*Epicypta* sp. 11	0	0	1	0	0
Mycetophilidae	Mycetophilinae	*Epicypta* sp. 12	5	0	1	0	0
Mycetophilidae	Mycetophilinae	*Epicypta* sp. 13	0	0	0	0	0
Mycetophilidae	Mycetophilinae	*Epicypta* sp. 14	3	2	2	0	0
Mycetophilidae	Mycetophilinae	*Epicypta* sp. 15	0	1	0	0	0
Mycetophilidae	Mycetophilinae	*Epicypta* sp. 16	0	1	0	0	0
Mycetophilidae	Mycetophilinae	*Epicypta* sp. 17	48	2	1	0	0
Mycetophilidae	Mycetophilinae	*Epicypta* sp. 18	10	1	0	0	0
Mycetophilidae	Mycetophilinae	*Epicypta* sp. 19	1	0	0	0	0
Mycetophilidae	Mycetophilinae	*Epicypta* sp. 20	1	0	0	0	0

Mycetophilidae	Mycetophilinae	*Epicypta* sp. 21	2	0	0	0	0
Mycetophilidae	Mycetophilinae	*Epicypta* sp. 22	3	0	0	0	0
Mycetophilidae	Mycetophilinae	*Epicypta* sp. 23	1	0	0	0	0
Mycetophilidae	Mycetophilinae	*Epicypta* sp. 24	1	0	0	0	0
Mycetophilidae	Mycetophilinae	*Epicypta* sp. 25	1	0	0	0	0
Mycetophilidae	Mycetophilinae	*Epicypta* sp. 26	1	0	0	0	0
Mycetophilidae	Mycetophilinae	*Epicypta* sp. 27	2	0	0	0	0
Mycetophilidae	Mycetophilinae	*Epicypta* sp. 28	1	0	0	0	0
Mycetophilidae	Mycetophilinae	*Epicypta* sp. 29	1	0	0	0	0
Mycetophilidae	Mycetophilinae	*Epicypta unident. females*	18	0	0	0	0
Mycetophilidae	Mycetophilinae	*Exechiopsis* sp. 2	1	0	0	0	0
Mycetophilidae	Mycetophilinae	*Mycetophila* sp. 2	1	0	0	0	0
Mycetophilidae	Mycetophilinae	*Neallodia* sp. 1	1	0	0	0	0
Mycetophilidae	Mycetophilinae	*Neallodia* sp. 2	1	0	0	0	0
Mycetophilidae	Mycetophilinae	*Rymosia damascenoi* Lane	1	0	0	0	0
Mycetophilidae	Mycetophilinae	*Zygomyia* sp. 1	4	0	0	0	0
Mycetophilidae	Mycetophilinae	*Zygomyia* sp. 2	3	0	0	0	0
Scatopsidae	Scatopsinae	*Neorhegmoclemina* sp. 1	1	2	0	1	0
Scatopsidae	Scatopsinae	*Neorhegmoclemina* sp. 2	0	1	0	0	0
Scatopsidae	Scatopsinae	*Neorhegmoclemina* ? sp. 1	0	1	0	0	0
Scatopsidae	Scatopsinae	*Thripomorpha* sp. 1	2	0	0	0	0
Scatopsidae	Scatopsinae	*Brahemyia* sp. 1	1	1	1	0	0
Scatopsidae	Scatopsinae	*Brahemyia* sp. 2	1	0	0	0	0
Scatopsidae	Scatopsinae	*Aztecatopse* sp. 1	0	1	0	0	0
Rhagionidae	Chrysopilinae	*Chrysopilus* sp. 1	1	0	0	0	0
Rhagionidae	Chrysopilinae	*Chrysopilus* sp. 2	1	0	0	0	0
Rhagionidae	Chrysopilinae	*Chrysopilus* sp. 3	0	0	0	1	0
Tabanidae	Pangoniinae	*Fidena pseudoaurimaculata*	1	2	1	1	1
Tabanidae	Chrysopsinae	*Chrysops variegatus*	0	2	0	1	0
Tabanidae	Chrysopsinae	*Chrysops cf incisus*	0	0	0	1	0
Tabanidae	Tabaninae	*Stypommisa glandicolor*	39	2	0	1	0
Tabanidae	Tabaninae	*Catachlorops rubiginosus*	1	0	0	0	0
Tabanidae	Tabaninae	*Leucotabanus exaestuan*	1	0	0	0	0
Tabanidae	Tabaninae	*Dichelacera cervicornis*	1	0	0	0	0
Tabanidae	Tabaninae	*Tabanus cf trivittatus*	4	1	0	0	0
Tabanidae	Tabaninae	*Tabanus antarcticus*	1	0	0	0	0
Tabanidae	Tabaninae	*Tabanus cf occidentalis*	1	0	0	0	0
Tabanidae	Tabaninae	*Tabanus nematocallus*	4	0	0	0	0
Tabanidae	Tabaninae	*Tabanus* sp1	3	0	0	0	0
Tabanidae	Tabaninae	*Tabanus fortis*	1	1	0	0	0
Tabanidae	Tabaninae	*Chlorotabanus inanis*	1	1	1	0	0
Tabanidae	Tabaninae	*Stibasoma currani*	0	1	0	0	1
Tabanidae	Tabaninae	*Acanthocera gorayebi*	0	3	3	0	0
Tabanidae	Tabaninae	*Acanthocera marginalis*	0	1	0	0	0
Tabanidae	Tabaninae	*Diachlorus xynus*	0	5	4	0	1
Tabanidae	Tabaninae	*Philipotabanus stigmaticalis*	0	0	0	1	1
Stratiomyidae	Beridinae	*Arcuavena* sp. 1	1	0	0	0	0
Stratiomyidae	Beridinae	*Oplacantha* sp. 1	0	1	1	0	0
Stratiomyidae	Clitellariinae	*Cyphomyia unicolor*	2	0	0	0	0
Stratiomyidae	Clitellariinae	*Cyphomyia* sp. 1	0	0	0	1	0
Stratiomyidae	Clitellariinae	*Cyphomyia* sp. 2	0	1	0	0	0
Stratiomyidae	Clitellariinae	*Cyphomyia* sp. 3	1	0	0	0	0
Stratiomyidae	Clitellariinae	*Diaphorostylus* sp. 1	1	0	0	0	0
Stratiomyidae	Clitellariinae	*Euryneura* sp. 1	0	0	1	0	0
Stratiomyidae	Clitellariinae	*Euryneura* sp. 2	1	0	1	0	0
Stratiomyidae	Hermetiinae	*Hermetia flavipes*	0	0	2	0	0
Stratiomyidae	Hermetiinae	*Hermetia illucens*	0	0	0	1	0
Stratiomyidae	Hermetiinae	*Hermetia pulchra*	0	2	0	0	0
Stratiomyidae	Pachygastrinae	Artemita sp. 1	0	0	0	0	1
Stratiomyidae	Pachygastrinae	*Brachyodina dorsata*	1	2	0	0	0
Stratiomyidae	Pachygastrinae	*Cyclotaspis* sp. 1	2	0	0	0	0
Stratiomyidae	Pachygastrinae	*Eidalimus* sp. 1	4	0	0	0	0
Stratiomyidae	Pachygastrinae	*Eidalimus* sp. 2	3	0	0	0	0

Stratiomyidae	Pachygastrinae	*Gowdeyana* sp. 1	0	0	1	0	0
Stratiomyidae	Pachygastrinae	*Lyprotemyia* [or *Pachygaster*] sp. 1	0	0	2	0	0
Stratiomyidae	Pachygastrinae	*Manotes* sp. 1	0	0	1	0	0
Stratiomyidae	Pachygastrinae	*Proegmenomyia metallica*	1	0	0	0	0
Stratiomyidae	Sarginae	*Merosargus gracilis*	13	0	0	0	0
Stratiomyidae	Sarginae	*Merosargus incanus*	1	0	0	0	0
Stratiomyidae	Sarginae	*Merosargus nebulifer*	1	1	0	0	0
Stratiomyidae	Sarginae	*Merosargus pictipes*	8	0	1	0	0
Stratiomyidae	Sarginae	*Merosargus stamineus*	1	3	0	0	0
Stratiomyidae	Sarginae	*Merosargus tripartitus*	1	0	0	0	0
Stratiomyidae	Sarginae	*Merosargus* sp. 1	0	0	1	0	0
Stratiomyidae	Sarginae	*Ptecticus* sp. 1	1	0	0	0	0
Stratiomyidae	Sarginae	*Sargus brasiliensis*	0	5	0	0	0
Stratiomyidae	Sarginae	*Sargus fasciatus*	5	0	0	0	0
Stratiomyidae	Sarginae	*Sargus* sp. 1	1	0	0	0	0
Therevidae	-	*Ataenogera brevicornis*	0	0	0	5	0
Bombyliidae	Anthracinae	*Exoprosopa* sp. 1	0	1	0	1	0
Empididae	Empidinae	*Empis* sp. 1	6	8	13	63	7
Empididae	Empidinae	*Empis* sp. 2	1	0	0	0	0
Empididae	Empidinae	*Empis* sp. 3	0	0	0	1	0
Empididae	Empidinae	*Macrostomus albicaudatus*	20	5	0	0	0
Empididae	Empidinae	*Macrostomus cervicicauda*	2	0	3	4	0
Empididae	Empidinae	*Macrostomus manauara*	2	4	0	0	0
Empididae	Empidinae	*Macrostomus* sp. 1	4	0	0	0	0
Empididae	Empidinae	*Macrostomus* sp. 2	3	0	0	3	0
Empididae	Empidinae	*Macrostomus* sp. 3	0	1	0	0	0
Empididae	Empidinae	*Macrostomus ferrugineus*	1	0	0	0	0
Empididae	Empidinae	*Macrostomus flavus*	1	1	0	0	0
Empididae	Empidinae	*Macrostomus cysticercus*	3	0	0	0	0
Empididae	Empidinae	*Porphyrochroa* sp. 1	1	2	1	4	0
Empididae	Empidinae	*Porphyrochroa* sp. 2	9	4	5	0	0
Empididae	Empidinae	*Porphyrochroa* sp. 3	0	2	0	0	0
Empididae	Empidinae	*Porphyrochroa* sp. 4	0	0	1	0	0
Empididae	Empidinae	*Lamprempis* sp. 1	0	1	0	0	0
Empididae	Empidinae	*Lamprempis* sp. 2	2	0	0	0	0
Empididae	Empidinae	*Lamprempis* sp. 3	0	0	0	1	1
Empididae	Empidinae	*Lamprempis* sp. 4	1	0	0	0	0
Empididae	Empidinae	*Opeatocerata* sp. 1	0	1	0	0	0
Empididae	Empidinae	*Opeatocerata* sp. 2	0	1	0	0	0
Empididae	Empidinae	*Hilara* sp. 1	1	0	0	0	0
Empididae	Gen. nov. A	Gen. nov. A sp. 1	0	0	6	3	1
Dolichopodidae	Achalcinae	*Xanthina* sp. 1	3	0	0	0	0
Dolichopodidae	Achalcinae	*Xanthina* sp. 2	1	1	3	0	0
Dolichopodidae	Achalcinae	*Xanthina* sp. 3	0	1	0	0	0
Dolichopodidae	Achalcinae	*Xanthina* sp. f#	4	1	2	0	0
Dolichopodidae	Diaphorinae	*Chrysotus* sp. 1	78	1	16	9	30
Dolichopodidae	Diaphorinae	*Chrysotus* sp. 2	17	5	3	11	1
Dolichopodidae	Diaphorinae	*Chrysotus* sp. 3	1	0	1	3	0
Dolichopodidae	Diaphorinae	*Chrysotus* sp. 4	6	5	8	21	21
Dolichopodidae	Diaphorinae	*Chrysotus* sp. 5	0	0	1	1	3
Dolichopodidae	Diaphorinae	*Chrysotus* sp. 6	1	1	1	8	0
Dolichopodidae	Diaphorinae	*Chrysotus* sp. 7	1	0	0	0	0
Dolichopodidae	Diaphorinae	*Chrysotus* sp. 8	0	0	0	2	0
Dolichopodidae	Diaphorinae	*Chrysotus* sp. 9	0	0	0	4	0
Dolichopodidae	Diaphorinae	*Chrysotus* sp. 10	1	0	3	5	0
Dolichopodidae	Diaphorinae	*Chrysotus* sp. 11	0	0	1	3	4
Dolichopodidae	Diaphorinae	*Chrysotus* sp. 12	1	0	6	51	8
Dolichopodidae	Diaphorinae	*Chrysotus* sp. 13	1	0	1	0	0
Dolichopodidae	Diaphorinae	*Chrysotus* sp. 14	2	0	1	11	2
Dolichopodidae	Diaphorinae	*Chrysotus* sp. 15	0	0	1	0	0
Dolichopodidae	Diaphorinae	*Chrysotus* spp. 12-15 f#	4	0	6	63	2
Dolichopodidae	Diaphorinae	*Chrysotus spp. f#*	0	1	0	1	1
Dolichopodidae	Diaphorinae	*Diaphorus* sp. 1	0	0	0	0	1

Dolichopodidae	Diaphorinae	*Lyroneurus* sp. 1	0	0	0	0	6
Dolichopodidae	Diaphorinae	*Lyroneurus* sp. 2	0	0	9	50	19
Dolichopodidae	Dolichopodinae	*Paraclius* sp. 1	3	5	4	2	1
Dolichopodidae	Dolichopodinae	*Paraclius* sp. 2	2	3	2	0	0
Dolichopodidae	Dolichopodinae	*Paraclius* sp. 3	0	0	1	0	0
Dolichopodidae	Dolichopodinae	*Paraclius* sp. 4	0	0	0	1	0
Dolichopodidae	Medeterinae	*Dominicomyia* sp. 1	0	2	9	7	1
Dolichopodidae	Medeterinae	*Dominicomyia* sp. 2	0	13	1	0	1
Dolichopodidae	Medeterinae	*Dominicomyia* sp. 3	0	0	0	3	0
Dolichopodidae	Medeterinae	*Medetera* sp. 1	3	1	2	0	4
Dolichopodidae	Medeterinae	*Medetera* sp. 2	3	0	2	0	0
Dolichopodidae	Medeterinae	*Medetera* sp. 3	0	0	6	0	1
Dolichopodidae	Medeterinae	*Medetera* sp. 4	2	0	2	1	0
Dolichopodidae	Medeterinae	*Medetera* sp. 5	0	3	5	7	1
Dolichopodidae	Medeterinae	*Medetera* sp. 6	0	2	1	0	0
Dolichopodidae	Medeterinae	*Medetera* sp. 7	0	0	2	1	0
Dolichopodidae	Medeterinae	*Medetera* sp. 8	0	0	1	0	0
Dolichopodidae	Medeterinae	*Medetera* sp. 9	0	0	1	0	0
Dolichopodidae	Medeterinae	*Medetera* sp. 10	0	0	1	0	0
Dolichopodidae	Medeterinae	*Medetera* sp. 11	0	0	0	1	0
Dolichopodidae	Medeterinae	*Medetera* sp. 12	0	0	0	1	0
Dolichopodidae	Medeterinae	*Medetera* sp. 13	0	0	0	3	0
Dolichopodidae	Medeterinae	*Medetera* spp. f#	1	0	0	4	0
Dolichopodidae	Medeterinae	*Micromedetera* ? sp. 1	3	6	0	16	1
Dolichopodidae	Medeterinae	*Thrypticus* sp. 1	0	0	0	0	1
Dolichopodidae	Medeterinae	*Systenus* sp. 1	0	0	1	1	0
Dolichopodidae	Medeterinae	*Systenus* sp. 2	0	0	0	2	0
Dolichopodidae	Medeterinae	*Systenus* sp. 3	0	0	1	0	0
Dolichopodidae	Medeterinae	*Systenus* spp. f#	0	0	2	0	0
Dolichopodidae	Medeterinae	New genus 1 sp. 1	0	1	0	0	0
Dolichopodidae	Neurigoninae	*Coeloglutus* sp 1.	0	0	0	3	0
Dolichopodidae	Neurigoninae	*Dactylomyia* sp. 1	0	0	1	0	0
Dolichopodidae	Neurigoninae	*Dactylomyia* sp. 2	0	0	0	0	1
Dolichopodidae	Neurigoninae	*Neurigona* sp. 1	1	0	0	0	0
Dolichopodidae	Neurigoninae	*Neurigona* sp. 2	1	0	0	0	0
Dolichopodidae	Neurigoninae	*Neurigona* sp. 3	1	0	0	0	0
Dolichopodidae	Neurigoninae	*Neurigona* spp. f#	1	0	1	0	0
Dolichopodidae	Neurigoninae	*Viridigona* sp. 1	1	0	0	0	0
Dolichopodidae	Neurigoninae	*Viridigona* sp. 2	0	1	0	0	0
Dolichopodidae	Neurigoninae	*Viridigona* sp. 3	0	0	1	0	0
Dolichopodidae	Neurigoninae	*Viridigona* sp. 4	0	0	1	1	0
Dolichopodidae	Neurigoninae	*Viridigona spp. f#*	3	0	0	0	0
Dolichopodidae	Sciapodinae	*Amblypsilopus* sp. 1	0	2	15	11	38
Dolichopodidae	Sciapodinae	*Condylostylus* sp. 1	8	0	9	1	2
Dolichopodidae	Sciapodinae	*Condylostylus* sp. 2	1	0	10	4	2
Dolichopodidae	Sciapodinae	*Condylostylus* sp. 3	5	0	1	0	1
Dolichopodidae	Sciapodinae	*Condylostylus* sp. 4	3	1	1	1	2
Dolichopodidae	Sciapodinae	*Condylostylus* sp. 5	0	0	0	1	0
Dolichopodidae	Sciapodinae	*Condylostylus spp. f#*	2	0	0	0	1
Dolichopodidae	Sciapodinae	*Mesorhaga* sp. 1	0	0	0	0	1
Dolichopodidae	Sciapodinae	New genus 2 sp. 1	0	1	3	0	0
Dolichopodidae	Sciapodinae	New genus 2 spp. f#	2	2	5	2	0
Dolichopodidae	Sympycninae	*Sympycnus* sp. 1	0	5	0	0	0
Dolichopodidae	incertae sedis	*Somillus* sp. 1	3	0	0	0	0
Dolichopodidae	incertae sedis	*Symbolia* sp. 1	7	1	6	0	4
Dolichopodidae	incertae sedis	*Symbolia* sp. 2	2	2	16	1	3
Dolichopodidae	incertae sedis	*Symbolia* sp. 3	1	0	0	0	0
Dolichopodidae	incertae sedis	*Symbolia* sp. 4	0	0	0	0	1
Dolichopodidae	incertae sedis	*Symbolia spp. f#*	3	8	15	1	1
Platypezidae	Microsaniinae	*Microsania* sp. 1	0	0	0	2	0
Phoridae	Chonocephalinae	*Chonocephalus*	4	1	0	0	0
Phoridae	Phorinae	*Conicera*	1	0	0	0	0
Phoridae	Phorinae	*Coniceromyia*	9	92	2	2	0

Phoridae	Phorinae	*Dohrniphora*	37	6	0	8	45
Phoridae	Phorinae	*Neopleurophora*	1	0	0	0	0
Phoridae	Phorinae	*Chaetocnemistoptera*	3	0	0	0	0
Phoridae	Metopininae	*Acanthophorides*	0	0	0	0	1
Phoridae	Metopininae	*Allochaeta*	0	20	27	49	2
Phoridae	Metopininae	*Apocephalus*	609	69	8	20	5
Phoridae	Metopininae	*Apodicrania*	4	15	40	140	48
Phoridae	Metopininae	*Auxanommatidia*	0	0	0	2	0
Phoridae	Metopininae	*Beckerina*	1	1	0	0	0
Phoridae	Metopininae	*Calamiscus*	0	6	0	0	0
Phoridae	Metopininae	*Ceratoconus*	0	0	0	2	0
Phoridae	Metopininae	*Chaetaspidia*	0	1	0	0	0
Phoridae	Metopininae	*Cremersia*	1	0	0	0	0
Phoridae	Metopininae	*Dacnophora*	3	0	0	1	0
Phoridae	Metopininae	*Diocophora*	0	9	20	64	13
Phoridae	Metopininae	*Eibesfeldtphora*	1	2	0	0	0
Phoridae	Metopininae	*Gymnophora*	2	0	0	0	0
Phoridae	Metopininae	*Megaselia*	1013	388	52	242	80
Phoridae	Metopininae	*Melaloncha*	0	3	1	0	0
Phoridae	Metopininae	*Metopina* -group genera	287	27	2	33	2
Phoridae	Metopininae	*Myrmosicarius*	1	1	0	0	0
Phoridae	Metopininae	New genus 1	0	12	0	12	1
Phoridae	Metopininae	New genus 2	0	1	0	0	0
Phoridae	Metopininae	New genus 3	0	0	1	0	0
Phoridae	Metopininae	New genus 4	0	0	0	1	0
Phoridae	Metopininae	New genus 5	0	0	1	0	0
Phoridae	Metopininae	New genus 6	0	0	0	0	1
Phoridae	Metopininae	New genus 7	0	0	1	0	0
Phoridae	Metopininae	New genus 8	0	0	0	1	0
Phoridae	Metopininae	New genus 9	0	0	0	0	4
Phoridae	Metopininae	New genus 10	0	12	1	12	0
Phoridae	Metopininae	*Phalacrotophora*	0	1	1	0	0
Phoridae	Metopininae	*Phymatopterella*	4	1	0	0	0
Phoridae	Metopininae	*Physoptera*	0	1	1	0	1
Phoridae	Metopininae	*Platydipteron*	0	1	3	9	1
Phoridae	Metopininae	*Pseudacteon*	6	36	11	61	27
Phoridae	Metopininae	*Pseudohypocera*	0	0	2	1	7
Phoridae	Metopininae	*Rhynchophoromyia*	2	5	0	1	0
Phoridae	Metopininae	*Styletta*	0	1	0	0	0
Phoridae	Metopininae	*Syneura*	0	0	1	9	0
Phoridae	Metopininae	*Tabelliphora*	0	1	2	52	0
Phoridae	Metopininae	*Woodiphora*	0	2	0	0	1
Phoridae	Metopininae	*Xanionotum*	0	0	0	1	0
Pipunculidae		*Amazunculus* sp. nov.	0	1	0	0	0
Pipunculidae		*Basileunculus* sp.	0	0	0	1	0
Pipunculidae		*Cephalosphaera miriamae*	0	1	0	0	0
Pipunculidae		*Elmohardyia* sp 1	1	0	0	0	0
Pipunculidae		*Elmohardyia* sp. nov. (sp. 2)	0	0	0	1	0
Pipunculidae		*Eudorylas fortis*	0	0	1	1	0
Pipunculidae		*Eudorylas* sp. 1	0	1	0	0	0
Pipunculidae		*Eudorylas* sp. 2	0	0	1	0	0
Pipunculidae		*Eudorylas* sp. 3	0	0	1	0	0
Pipunculidae		*Eudorylas* sp. 4	0	0	0	1	0
Pipunculidae		*Eudorylas* sp. 5	0	0	0	1	0
Pipunculidae		Gen. nov. 1 sp. n. 1	1	0	0	0	0
Pipunculidae		Gen. nov. 2 sp. n. 1	0	1	0	0	0
Pipunculidae		Gen. nov. 3 sp. n. 1	0	0	0	1	0
Syrphidae	Eristalinae	*Alipumilio aff. femoratus*	0	1	0	0	0
Syrphidae	Eristalinae	*Copestylum* sp. 1	0	0	1	1	0
Syrphidae	Eristalinae	*Palpada scutellaris*	0	1	0	0	0
Syrphidae	Syrphinae	*Hybobathus* sp.	0	0	1	0	0
Syrphidae	Syrphinae	*Fragosa obliquus*	0	0	0	1	1
Syrphidae	Syrphinae	*Fragosa* sp. 1 (*rugosifrons* species group)	0	2	0	1	0

Syrphidae	Syrphinae	*Nuntianus* sp. 1	0	0	2	0	0
Syrphidae	Syrphinae	*Nuntianus* sp. 3	0	0	1	0	0
Syrphidae	Syrphinae	*Pelecinobaccha adspersa*	0	1	0	2	0
Syrphidae	Microdontinae	*Rhopalosyrphus* sp. 1	0	1	0	0	0
Syrphidae	Microdontinae	*Rhopalosyrphus* sp. 2 (sensu lato)	0	0	0	2	0
Syrphidae	Microdontinae	*Stipomorpha* sp. 1	0	0	1	0	0
Syrphidae	Microdontinae	*Stipomorpha* sp. 2	0	0	0	1	0
Syrphidae	Microdontinae	*Surimyia rolanderi*	0	0	3	0	0
Aulacigastridae		Aulacigaster sp. 1	1	0	0	0	0
Chloropidae	Oscinellinae	Conioscinella sp. 1	0	2	0	6	0
Chloropidae	Oscinellinae	Conioscinella sp. 2	0	1	0	1	0
Chloropidae	Oscinellinae	Conioscinella sp. 3	0	7	0	0	0
Chloropidae	Oscinellinae	Conioscinella sp. 4	4	0	0	0	0
Chloropidae	Oscinellinae	Fiebrigella sp. 1	0	0	0	1	0
Chloropidae	Oscinellinae	Fiebrigella sp. 2	0	0	0	1	0
Chloropidae	Oscinellinae	Gaurax ?sp. 2	0	0	0	0	1
Chloropidae	Oscinellinae	Gaurax sp. 1	2	0	0	0	0
Chloropidae	Oscinellinae	Gaurax sp. 2	0	0	1	0	0
Chloropidae	Oscinellinae	Gaurax sp. 3	0	1	0	0	0
Chloropidae	Oscinellinae	Gaurax sp. 4	0	2	0	0	0
Chloropidae	Oscinellinae	?Gaurax sp. 4	0	1	0	0	0
Chloropidae	Oscinellinae	Gaurax sp. 5	0	0	0	2	0
Chloropidae	Oscinellinae	*Metasiphonella magnifica*	0	0	4	10	0
Chloropidae	Oscinellinae	Notaulacella sp. 1	8	1	0	0	0
Chloropidae	Oscinellinae	Notaulacella sp. 2	2	0	0	0	0
Chloropidae	Oscinellinae	Olcella sp. 1	1	0	0	0	0
Chloropidae	Oscinellinae	Onychaspidium sp.	0	0	1	0	0
Chloropidae	Oscinellinae	*Pseudogaurax ?mexoculatus*	0	0	1	0	0
Chloropidae	Oscinellinae	*Pseudogaurax ?paratolmos*	1	0	0	0	0
Chloropidae	Oscinellinae	*Pseudogaurax nigrolineatus*	0	1	0	0	0
Chloropidae	Oscinellinae	*Pseudogaurax nigromaculatus*	0	1	0	0	0
Chloropidae	Oscinellinae	*Pseudogaurax souzalopesi*	0	1	0	0	0
Chloropidae	Oscinellinae	Pseudogaurax sp. 1	1	0	0	0	0
Chloropidae	Oscinellinae	*Pseudogaurax trilineatus*	0	0	0	1	0
Chloropidae	Oscinellinae	Rhopalopterum sp. 1	2	0	0	0	0
Chloropidae	Oscinellinae	Tricimba sp. 1	1	5	0	0	0
Chloropidae	Oscinellinae	Tricimba sp. 2	0	3	0	0	0
Chloropidae	Oscinellinae	Oscinellinae gen. indet. sp.	0	1	0	0	0
Clusiidae	Sobarocephainae	Sobarocephala sp. 1	0	0	0	1	0
Clusiidae	Sobarocephainae	Sobarocephala sp. 2	0	0	0	1	0
Clusiidae	Sobarocephainae	Sobarocephala sp. 3	0	0	0	0	1
Clusiidae	Sobarocephainae	Sobarocephala sp. 4	2	0	0	0	0
Clusiidae	Sobarocephainae	Sobarocephala sp. 5	0	0	0	1	0
Clusiidae	Sobarocephainae	Sobarocephala sp. 6	0	0	0	1	0
Clusiidae	Sobarocephainae	Sobarocephala sp. 7	0	0	0	1	0
Clusiidae	Sobarocephainae	Sobarocephala sp. 8	0	0	0	2	0
Clusiidae	Sobarocephainae	Sobarocephala sp. 9	0	0	0	1	0
Clusiidae	Sobarocephainae	Sobarocephala sp. 10	0	2	0	0	0
Clusiidae	Sobarocephainae	Sobarocephala sp. 11	0	2	0	0	0
Clusiidae	Sobarocephainae	Sobarocephala sp. 12	0	1	0	0	0
Clusiidae	Sobarocephainae	Sobarocephala sp. 13	0	1	0	0	0
Clusiidae	Sobarocephainae	Sobarocephala sp. 14	0	1	0	0	0
Clusiidae	Sobarocephainae	Sobarocephala sp. 15	0	1	0	0	0
Clusiidae	Sobarocephainae	Sobarocephala sp. 16	0	1	0	0	0
Conopidae	Stylogastrinae	Stylogaster sp. 1	2	0	0	0	0
Conopidae	Stylogastrinae	Stylogaster sp. 2	0	2	0	0	0
Conopidae	Stylogastrinae	Stylogaster sp. 3	0	0	0	2	0
Drosophilidae	Drosophilinae	Chymomyza sp. 1	1	1	0	0	0
Drosophilidae	Drosophilinae	*Cladochaeta atlantica*	0	1	0	0	0
Drosophilidae	Drosophilinae	Cladochaeta sp. 1	1	0	0	0	0
Drosophilidae	Drosophilinae	Cladochaeta sp. 2	1	0	0	1	0
Drosophilidae	Drosophilinae	Cladochaeta sp. 3	0	1	1	0	0
Drosophilidae	Drosophilinae	Cladochaeta sp. 5	0	0	1	0	0

Drosophilidae	Drosophilinae	Cladochaeta sp. 6	0	0	1	0	0
Drosophilidae	Drosophilinae	*Diathoneura tesselata*	3	0	0	0	0
Drosophilidae	Drosophilinae	Diathoneura sp. 1	1	0	0	0	0
Drosophilidae	Drosophilinae	Diathoneura sp. 2	1	0	0	0	0
Drosophilidae	Drosophilinae	Diathoneura sp. 3	0	2	1	0	0
Drosophilidae	Drosophilinae	Diathoneura sp. 4	0	1	0	0	0
Drosophilidae	Drosophilinae	Diathoneura sp. 5	0	1	0	0	0
Drosophilidae	Drosophilinae	Diathoneura sp. 6	0	0	0	1	0
Drosophilidae	Drosophilinae	*Drosophila aff. busckii*	1	0	0	0	0
Drosophilidae	Drosophilinae	*Drosophila aff. immigrans*	1	1	0	0	0
Drosophilidae	Drosophilinae	Drosophila sp. 1 (tripunctata group)	5	0	0	1	0
Drosophilidae	Drosophilinae	Drosophila sp. 2	2	0	0	0	0
Drosophilidae	Drosophilinae	Drosophila sp. 3	1	0	0	0	0
Drosophilidae	Drosophilinae	Drosophila sp. 4	4	0	0	0	0
Drosophilidae	Drosophilinae	Drosophila sp. 5	0	1	2	7	1
Drosophilidae	Drosophilinae	Drosophila sp. 6	0	0	0	1	0
Drosophilidae	Drosophilinae	Drosophila sp. 7	0	0	0	1	0
Drosophilidae	Drosophilinae	Drosophila sp. 8	0	0	0	1	0
Drosophilidae	Drosophilinae	Microdrosophila sp. 1	0	2	1	0	0
Drosophilidae	Drosophilinae	Microdrosophila sp. 2	0	2	0	0	0
Drosophilidae	Drosophilinae	Microdrosophila sp. 3	0	2	1	0	0
Drosophilidae	Drosophilinae	Microdrosophila sp. 4	0	0	1	0	0
Drosophilidae	Drosophilinae	Microdrosophila sp. 5	0	0	0	1	0
Drosophilidae	Drosophilinae	*Microdrosophila* spp n id (females)	2	2	0	2	0
Drosophilidae	Drosophilinae	Mycodrosophila sp. 1	0	1	0	0	0
Drosophilidae	Drosophilinae	*Neotanygastrella aff. brasiliensis*	3	1	3	2	0
Drosophilidae	Drosophilinae	*Neotanygastrella aff. chymomyzoides*	1	0	0	0	0
Drosophilidae	Drosophilinae	Zygothrica sp. 1	0	0	0	1	0
Drosophilidae	Drosophilinae	Zygothrica sp. 2	0	0	0	1	0
Drosophilidae	Drosophilinae	Genero não identicado	0	0	0	1	0
Drosophilidae	Steganinae	Amiota sp. 1	0	0	1	0	0
Drosophilidae	Steganinae	Leucophenga sp. 1	0	0	0	0	1
Drosophilidae	Steganinae	*Pseudiastata pseudococcivora*	0	1	0	0	1
Drosophilidae	Steganinae	Rhinoleucophenga sp. 1	1	0	0	0	0
Drosophilidae	Steganinae	Rhinoleucophenga sp. 2	0	1	0	0	0
Drosophilidae	Steganinae	Rhinoleucophenga sp. 4	0	0	1	3	0
Drosophilidae	Steganinae	Rhinoleucophenga sp. 3	2	0	2	0	0
Drosophilidae	Steganinae	Rhinoleucophenga sp. 5	0	0	1	2	0
Drosophilidae	Steganinae	Rhinoleucophenga sp. 6	0	0	0	1	1
Drosophilidae	Steganinae	Rhinoleucophenga sp. 7	0	0	0	5	7
Drosophilidae	Steganinae	Rhinoleucophenga sp. 8	0	0	0	1	0
Drosophilidae	Steganinae	Rhinoleucophenga sp. 9	0	0	0	1	0
Drosophilidae	Steganinae	Rhinoleucophenga sp. 10	0	0	0	0	1
Drosophilidae	Steganinae	Stegana sp. 1	1	0	0	0	0
Drosophilidae	Steganinae	Stegana sp. 2	0	0	1	0	0
Heleomyzidae	Rhinotorinae	Neorhinotora sp. 1	0	1	0	0	0
Inbiomyiidae		Inbiomyia sp. 1	2	0	0	0	0
Lauxaniidae	Lauxaniinae	Deceia spn 1	0	0	1	0	0
Lauxaniidae	Lauxaniinae	Marmarodeceia marmorata	0	0	0	1	0
Lauxaniidae	Lauxaniinae	Neoxangelina sp 1	4	7	11	2	0
Lauxaniidae	Lauxaniinae	Neoxangelina sp 2	3	2	0	3	0
Lauxaniidae	Lauxaniinae	Neoxangelina sp 3	0	1	0	0	0
Lauxaniidae	Lauxaniinae	Oncodometopus umbrosus	0	4	1	2	0
Lauxaniidae	Lauxaniinae	Oncodometopus sp 1	0	5	14	20	0
Lauxaniidae	Lauxaniinae	Oncodometopus sp 2	0	16	16	51	9
Lauxaniidae	Lauxaniinae	Oncodometopus sp 3	0	7	6	13	0
Lauxaniidae	Lauxaniinae	Oncodometopus sp 4	0	0	1	0	0
Lauxaniidae	Lauxaniinae	Oncodometopus sp 5	2	0	1	4	0
Lauxaniidae	Lauxaniinae	Oncodometopus sp 6	1	1	2	2	4
Lauxaniidae	Lauxaniinae	Oncodometopus sp 7	0	1	0	0	0
Lauxaniidae	Lauxaniinae	Physegenua? Sp 1	0	0	1	0	0
Lauxaniidae	Lauxaniinae	Physegenua? sp 2	0	0	4	0	0
Lauxaniidae	Lauxaniinae	Physoclypeus? sp 2	1	0	0	0	0

Lauxaniidae	Lauxaniinae	Poecilominettia brunneicosta?	0	0	0	0	1
Lauxaniidae	Lauxaniinae	Poecilominettia effosa group sp 1	0	0	3	0	0
Lauxaniidae	Lauxaniinae	Poecilominettia effosa group sp 2	0	0	1	0	0
Lauxaniidae	Lauxaniinae	Poecilominettia effosa group sp 3	0	1	0	0	0
Lauxaniidae	Lauxaniinae	Poecilominettia grata	0	0	0	3	0
Lauxaniidae	Lauxaniinae	Poecilominettia sp 3	0	1	2	1	1
Lauxaniidae	Lauxaniinae	Poecilominettia sp 4	0	0	0	2	0
Lauxaniidae	Lauxaniinae	Poecilominettia sp 5	0	0	0	2	0
Lauxaniidae	Lauxaniinae	Pseudogriphoneura sp 3	0	1	0	0	0
Lauxaniidae	Lauxaniinae	Siphonophysa sp 2	1	1	0	0	0
Lauxaniidae	Lauxaniinae	Siphonophysa sp 3	1	0	0	0	0
Lauxaniidae	Lauxaniinae	Scutominettia mallochi	1	0	0	0	0
Lauxaniidae	Lauxaniinae	Stenolauxania sp 1	1	1	0	0	0
Lauxaniidae	Lauxaniinae	Trivialia puella	0	0	0	1	0
Lauxaniidae	Lauxaniinae	Trivialia sp 4	1	1	0	0	0
Lauxaniidae	Lauxaniinae	Trivialia sp 5	0	1	0	0	0
Lauxaniidae	Lauxaniinae	Xenochaetina setitibia	0	0	1	0	0
Lauxaniidae	Lauxaniinae	Xenochaetina sp 1	0	0	0	1	0
Lauxaniidae	Lauxaniinae	Xenochaetina sp 2	0	1	2	1	0
Lauxaniidae	Lauxaniinae	Xenochaetina sp 6	0	0	1	0	0
Lauxaniidae	Lauxaniinae	Xenochaetina sp 7	2	1	1	2	1
Lauxaniidae	Lauxaniinae	Xenochaetina sp 8	0	0	1	2	0
Lauxaniidae	Lauxaniinae	Xenochaetina sp 9	0	0	1	0	0
Lauxaniidae	Lauxaniinae	Xenochaetina sp 10	1	0	0	0	0
Lauxaniidae	Lauxaniinae	Xenochaetina sp 11	1	1	0	2	0
Lauxaniidae	Lauxaniinae	Griphoneuromima sp 1	0	0	1	0	0
Lauxaniidae	Lauxaniinae	Meraina? sp 1	0	0	0	1	0
Lauxaniidae	Lauxaniinae	Undescribed genus A sp 1	0	0	1	0	0
Lauxaniidae	Lauxaniinae	Undescribed genus F sp 1	1	0	0	0	0
Lauxaniidae	Lauxaniinae	Undescribed genus nov sp 1	0	0	0	2	0
Micropezidae		Poecilotylus sp 1 R-group	1	0	0	0	0
Micropezidae		Poecilotylus sp 2_trifasciata group	1	0	1	1	0
Micropezidae		Poecilotylus sp 3	1	0	0	0	0
Micropezidae		*Grallipeza unimaculata*	2	3	1	1	0
Micropezidae		Cardiacephala sp 1	1	0	0	0	0
Micropezidae		Genus A *hyaloptera* (Hendel)	1	0	0	0	0
Micropezidae		Scipopus sp 1	0	2	1	0	0
Micropezidae		Genus cf *Paragrallomyia* sp 1	0	1	0	0	0
Micropezidae		Paragrallomyia sp 1	0	0	0	1	0
Milichiidae	Milichiinae	Milichia sp. 1	1	0	0	0	0
Milichiidae	Milichiinae	Milichia sp. 2	1	0	0	0	0
Milichiidae	Milichiinae	Milichiella sp. 1	1	0	0	1	0
Milichiidae	Milichiinae	Milichiella sp. 2	0	0	1	0	0
Milichiidae	Milichiinae	Milichiella sp. 3	0	0	0	1	0
Milichiidae	Milichiinae	Milichiella sp. 4	0	0	0	1	0
Milichiidae	Milichiinae	Pholeomyia sp. 1	1	0	0	0	0
Milichiidae	Milichiinae	Pholeomyia sp. 2	1	0	0	0	0
Milichiidae	Milichiinae	Pholeomyia sp. 3	1	0	0	0	0
Milichiidae	Milichiinae	Pholeomyia sp. 4	1	0	0	6	0
Milichiidae	Milichiinae	Pholeomyia sp. 5	0	1	0	0	0
Milichiidae	Milichiinae	Pholeomyia sp. 6	0	0	1	1	1
Milichiidae	Milichiinae	Pholeomyia sp. 7	0	0	2	1	0
Milichiidae	Milichiinae	Pholeomyia sp. 8	0	0	3	0	0
Milichiidae	Milichiinae	Pholeomyia sp. 9	0	0	0	0	0
Milichiidae	Milichiinae	Pholeomyia sp. 10	0	0	0	1	0
Milichiidae	Milichiinae	Pholeomyia sp. 11	0	0	0	1	0
Milichiidae	Milichiinae	Pholeomyia sp. 12	0	0	0	2	0
Milichiidae	Milichiinae	Pseudomilichia sp. 1	4	0	0	0	0
Milichiidae	Milichiinae	Desmometopa sp. 1	2	0	0	0	0
Milichiidae	Milichiinae	Desmometopa sp. 2	1	0	0	0	0
Milichiidae	Milichiinae	Leptometopa sp. 1	1	0	0	0	0
Milichiidae	Phyllomyzinae	Paramyia sp. 1	1	0	1	1	1
Milichiidae	Phyllomyzinae	Paramyia sp. 2	122	80	3	8	7

Milichiidae	Phyllomyzinae	Paramyia sp. 3	35	9	23	10	0
Milichiidae	Phyllomyzinae	Paramyia sp. 4	23	15	0	0	0
Milichiidae	Phyllomyzinae	Paramyia sp. 5	4	0	1	0	0
Milichiidae	Phyllomyzinae	Paramyia sp. 6	7	4	0	0	0
Milichiidae	Phyllomyzinae	Paramyia sp. 7	0	16	0	0	0
Milichiidae	Phyllomyzinae	Paramyia sp. 8	0	1	0	0	0
Milichiidae	Phyllomyzinae	Paramyia sp. 9	0	0	1	0	0
Milichiidae	Phyllomyzinae	Paramyia sp. 10	0	0	0	2	0
Milichiidae	Phyllomyzinae	Paramyia sp. 11	0	0	0	6	0
Milichiidae	Phyllomyzinae	Paramyia sp. 12	0	0	0	10	0
Milichiidae	Phyllomyzinae	Paramyia sp. 13	0	0	0	0	1
Milichiidae	Phyllomyzinae	Phyllomyza sp. 1	0	0	0	3	0
Milichiidae	Phyllomyzinae	Phyllomyza sp. 2	0	0	0	1	0
Neriidae		Glyphidops sp. 1	0	1	2	0	0
Neriidae		Glyphidops sp. 2	0	0	1	0	0
Odiniidae	Traginopinae	Helgreelia sp. 1	0	0	0	1	0
Odiniidae	Traginopinae	Inpauema sp. 1	0	0	0	1	0
Odiniidae	Odiniinae	Odinia sp. 1	0	1	1	4	0
Odiniidae	Odiniinae	Odinia sp. 2	0	1	3	1	0
Odiniidae	Odiniinae	Odinia sp. 3	0	0	0	1	1
Odiniidae	Odiniinae	Odinia sp. 4	0	0	1	1	0
Odiniidae	Odiniinae	Odinia sp. 5	2	0	0	0	0
Odiniidae	Traginopinae	Schildomyia sp. 1	0	0	0	0	1
Pseudopomyzidae		Rhinopomyzella sp. 1	0	0	0	0	1
Sepsidae	Sepsinae	*Archisepsis* sp	0	1	0	0	0
Sepsidae	Sepsinae	*Microsepsis armillata*	1	0	0	0	0
Sepsidae	Sepsinae	*Archisepsis excavata*	4	0	0	0	0
Anthomyiidae		*Phaonantho* sp	0	1	0	0	0
Muscidae	Cyrtoneurinae	*Cyrtoneuropsis conspersa* (Stein)	22	14	2	8	0
Muscidae	Cyrtoneurinae	*Cyrtoneuropsis multomaculata* (Stein)	0	1	0	5	1
Muscidae	Cyrtoneurinae	*Cyrtoneuropsis armipes* (Snyder)	0	0	0	1	0
Muscidae	Cyrtoneurinae	*Cyrtoneuropsis* sp 1	0	0	1	0	0
Muscidae	Cyrtoneurinae	*Cyrtoneuropsis* sp 2	0	0	1	0	1
Muscidae	Cyrtoneurinae	*Cyrtoneuropsis* sp 3	0	0	0	0	1
Muscidae	Cyrtoneurinae	*Cyrtoneuropsis* sp 4	0	0	0	1	0
Muscidae	Cyrtoneurinae	*Cyrtoneuropsis* sp 5	0	0	1	0	0
Muscidae	Cyrtoneurinae	*Cyrtoneuropsis* sp 6	0	0	0	1	0
Muscidae	Cyrtoneurinae	*Cyrtoneuropsis* sp 7	0	0	1	0	0
Muscidae	Cyrtoneurinae	*Cyrtoneuropsis* sp 8	0	0	0	1	0
Muscidae	Cyrtoneurinae	*Cyrtoneuropsis* sp 9	0	0	1	1	0
Muscidae	Cyrtoneurinae	*Cyrtoneuropsis* sp 10	0	0	1	0	0
Muscidae	Cyrtoneurinae	*Cyrtoneurina* sp 1 *	0	0	0	0	1
Muscidae	Cyrtoneurinae	*Cyrtoneurina* sp 2 *	0	0	0	0	1
Muscidae	Coenosiinae	*Neodexiopsis neoaustralis* Snyder	0	0	0	1	3
Muscidae	Coenosiinae	*Neodexiopsis emmesa* Malloch	6	0	1	9	16
Muscidae	Coenosiinae	*Neodexiopsis* sp 1	1	0	0	0	0
Muscidae	Coenosiinae	*Neodexiopsis* sp 2	2	0	0	0	0
Muscidae	Coenosiinae	*Neodexiopsis* sp 3	0	1	0	0	0
Muscidae	Coenosiinae	*Neodexiopsis* sp 4	0	0	1	0	0
Muscidae	Coenosiinae	*Neodexiopsis* sp 5	0	1	0	0	0
Muscidae	Coenosiinae	*Neodexiopsis sp 6*	0	0	0	0	1
Muscidae	Coenosiinae	*Neodexiopsis sp* 7	0	0	0	0	1
Muscidae	Coenosiinae	*Neodexiopsis* sp 8	0	0	0	0	1
Muscidae	Coenosiinae	*Neodexiopsis* sp 9	0	0	0	1	0
Muscidae	Coenosiinae	*Coenosia* sp 1	3	0	0	0	0
Muscidae	Coenosiinae	*Coenosia* sp 2	0	0	0	2	0
Muscidae	Coenosiinae	*Coenosia* sp 3	1	0	0	0	0
Muscidae	Coenosiinae	*Coenosia* sp 4	0	0	0	1	1
Muscidae	Coenosiinae	*Coenosia* sp 5	1	0	0	0	0
Muscidae	Coenosiinae	*Coenosia* sp 6	0	0	0	1	0
Muscidae	Coenosiinae	*Coenosia* sp 7	0	0	0	0	1
Muscidae	Coenosiinae	*Lispe serotina* Wulp	0	0	0	0	2
Muscidae	Coenosiinae	*Bithoracochaeta* sp *	3	0	0	0	0

Muscidae	Muscinae	*Stomopogon sp*	0	1	0	0	0
Muscidae	Muscinae	*Polietina* sp *	0	0	0	1	0
Muscidae	Muscinae	*Morellia* sp	0	1	0	0	0
Muscidae	Azeliinae	*Philornis* sp	0	1	0	0	0
Muscidae	Phaoninae	*Phaonia* sp	0	1	0	0	0
Muscidae	Phaoninae	*Dolichophaonia* sp *	0	1	0	0	0
Rhinophoridae		Bezzimyia sp. 1	1	0	0	0	0
Rhinophoridae		Undescribed genus sp. 1	1	0	0	0	0
Sarcophagidae	Sarcophaginae	*Blaesoxipha (Tephromyia) rimosa*	0	0	0	0	1
Sarcophagidae	Sarcophaginae	*Blaesoxipha* (*Tephromyia*) *tingomaria*	0	0	0	0	3
Sarcophagidae	Sarcophaginae	*Dexosarcophaga globulosa*	0	0	0	1	0
Sarcophagidae	Sarcophaginae	*Dexosarcophaga transita*	3	0	0	0	22
Sarcophagidae	Sarcophaginae	*Emdenimyia korytkowskii*	0	0	1	0	0
Sarcophagidae	Sarcophaginae	*Emdenimyia limai*	0	0	0	0	1
Sarcophagidae	Sarcophaginae	*Lepidodexia* (*Harpagopyga*) *albida*	1	0	0	0	0
Sarcophagidae	Sarcophaginae	*Lepidodexia* (*Notochaetisca*) sp.n. 1	0	1	0	0	0
Sarcophagidae	Sarcophaginae	*Lepidodexia* (*Notochaetisca*) sp.n. 2	0	0	0	0	1
Sarcophagidae	Sarcophaginae	*Orosarcophaga* sp.n. 1	0	0	0	1	0
Sarcophagidae	Sarcophaginae	*Retrocitomyia retrocita*	0	0	0	1	0
Sarcophagidae	Sarcophaginae	*Peckia (Sarcodexia) lambens*	0	0	0	0	2
Tachinidae	Dexiinae	Campylocheta sp. 1	0	14	4	8	1
Tachinidae	Dexiinae	Periscepsia sp. 1	0	0	1	0	0
Tachinidae	Dexiinae	Periscepsia sp. 2	0	0	0	0	1
Tachinidae	Dexiinae	*Zelia magna*	1	0	0	0	0
Tachinidae	Dexiinae	Zelia sp. 1	0	0	1	0	0
Tachinidae	Dexiinae	*Neozelia alini*	0	0	1	0	0
Tachinidae	Dexiinae	Billaea sp. 1	0	1	0	0	0
Tachinidae	Dexiinae	Billaea sp. 2	0	0	1	0	0
Tachinidae	Dexiinae	*Cordyligaster petiolata*	0	3	3	0	0
Tachinidae	Dexiinae	*Euoestrophasia aperta*	0	0	0	0	3
Tachinidae	Dexiinae	Cenosoma sp. 1	0	0	0	0	1
Tachinidae	Phasiinae	Xanthomelanopsis sp. 1	1	0	0	0	0
Tachinidae	Phasiinae	Euacaulona sp. 1	0	1	0	0	0
Tachinidae	Phasiinae	Unknown genus sp. 1	0	1	0	0	0
Tachinidae	Phasiinae	Strongygaster sp. 1	0	0	0	0	1
Tachinidae	Exoristinae	Acemya sp. 1	0	1	1	0	0
Tachinidae	Exoristinae	cf. Ceracia sp. 1	0	0	0	0	1
Tachinidae	Exoristinae	Anisia sp. 1	0	1	0	0	0
Tachinidae	Exoristinae	cf. Anisia sp. 1	0	0	2	0	25
Tachinidae	Exoristinae	cf. Anoxynops sp. 1	0	1	0	0	0
Tachinidae	Exoristinae	*Borgmeiermyia cf. rozeni*	18	23	0	1	0
Tachinidae	Exoristinae	*Borgmeiermyia cf. brasiliana*	0	3	7	0	0
Tachinidae	Exoristinae	Borgmeiermyia sp. 1	3	0	1	0	0
Tachinidae	Exoristinae	Borgmeiermyia sp. 2	7	0	0	0	0
Tachinidae	Exoristinae	Calodexia sp. 1	2	2	0	0	0
Tachinidae	Exoristinae	Chaetostigmoptera sp. 1	0	2	2	0	0
Tachinidae	Exoristinae	Blondelia sp. 1	0	0	1	0	1
Tachinidae	Exoristinae	Unknown genus sp. 1	0	0	1	0	0
Tachinidae	Exoristinae	Celatoria sp. 1	0	1	4	0	0
Tachinidae	Exoristinae	Celatoria sp. 2	0	2	7	6	16
Tachinidae	Exoristinae	Calolydella sp. 1	0	1	0	0	0
Tachinidae	Exoristinae	Calolydella sp. 2	0	0	0	8	0
Tachinidae	Exoristinae	cf. Eribella sp. 1	0	0	0	0	2
Tachinidae	Exoristinae	Erythromelana sp. 1	0	4	0	0	0
Tachinidae	Exoristinae	Erythromelana sp. 2	0	4	0	0	0
Tachinidae	Exoristinae	Erythromelana sp. 3	0	1	1	0	0
Tachinidae	Exoristinae	Erythromelana sp. 4	0	7	0	0	0
Tachinidae	Exoristinae	cf. Erythromelana sp. 1	0	0	0	1	0
Tachinidae	Exoristinae	aff. Erythromelana sp. 1	0	0	1	0	0
Tachinidae	Exoristinae	aff. Erythromelana sp. 2	0	0	0	0	1
Tachinidae	Exoristinae	aff. Erythromelana sp. 3	0	0	0	0	1
Tachinidae	Exoristinae	Eucelatoria sp. 1	0	1	0	0	0
Tachinidae	Exoristinae	Eucelatoria sp. 2	0	0	1	0	0

Tachinidae	Exoristinae	Eucelatoria sp. 3	0	0	1	0	0
Tachinidae	Exoristinae	Eucelatoria sp. 4	0	4	0	2	0
Tachinidae	Exoristinae	Eucelatoria sp. 5	0	0	0	1	0
Tachinidae	Exoristinae	Eucelatoria sp. 6	0	0	0	0	1
Tachinidae	Exoristinae	Eucelatoria sp. 7	0	0	1	0	0
Tachinidae	Exoristinae	Eucelatoria sp. 8	0	0	0	0	1
Tachinidae	Exoristinae	cf. Eucelatoria sp. 1	0	0	2	0	0
Tachinidae	Exoristinae	aff. Eucelatoria sp. 1	0	0	0	0	2
Tachinidae	Exoristinae	aff. Eucelatoria sp. 2	0	0	1	0	0
Tachinidae	Exoristinae	aff. Eucelatoria sp. 3	0	0	1	0	0
Tachinidae	Exoristinae	Euhalidaya sp. 1	0	0	2	0	0
Tachinidae	Exoristinae	Euhalidaya sp. 2	0	3	0	0	0
Tachinidae	Exoristinae	Euhalidaya sp. 3	0	0	0	0	1
Tachinidae	Exoristinae	Euhalidaya sp. 4	0	1	0	0	0
Tachinidae	Exoristinae	aff. Euhalidaya sp. 1	0	0	0	2	0
Tachinidae	Exoristinae	cf. Ischyrophaga sp. 1	0	2	0	0	0
Tachinidae	Exoristinae	cf. Ischyrophaga sp. 2	0	0	4	0	0
Tachinidae	Exoristinae	cf. Ischyrophaga sp. 3	0	0	1	0	0
Tachinidae	Exoristinae	aff. Ischyrophaga sp. 1	0	0	1	0	0
Tachinidae	Exoristinae	cf. Italispidea sp. 1	0	9	0	0	0
Tachinidae	Exoristinae	cf. Italispidea sp. 2	0	0	2	1	1
Tachinidae	Exoristinae	cf. Italispidea sp. 3	0	2	0	0	0
Tachinidae	Exoristinae	cf. Italispidea sp. 4	0	1	0	0	0
Tachinidae	Exoristinae	cf. Italispidea sp. 5	0	1	2	2	0
Tachinidae	Exoristinae	aff. Italispidea sp. 1	0	0	0	0	1
Tachinidae	Exoristinae	Lixophaga sp. 1	0	1	0	0	0
Tachinidae	Exoristinae	cf. Lixophaga sp. 1	0	1	0	0	0
Tachinidae	Exoristinae	Lydinolydella sp. 1	0	1	0	0	0
Tachinidae	Exoristinae	Lydinolydella sp. 2	0	1	0	0	0
Tachinidae	Exoristinae	Myiopharus sp. 1	0	0	0	0	2
Tachinidae	Exoristinae	Myiopharus sp. 2	0	0	3	0	0
Tachinidae	Exoristinae	aff. Myiopharus sp. 1	0	0	0	0	1
Tachinidae	Exoristinae	aff. Ophirion sp. 1	0	0	0	1	0
Tachinidae	Exoristinae	Phasmophaga sp. 1	0	0	0	0	3
Tachinidae	Exoristinae	Phyllophilopsis sp. 1	0	0	2	0	1
Tachinidae	Exoristinae	Phyllophilopsis sp. 2	0	0	0	0	1
Tachinidae	Exoristinae	Phyllophilopsis sp. 3	0	0	0	1	0
Tachinidae	Exoristinae	Phyllophilopsis sp. 4	0	0	0	0	2
Tachinidae	Exoristinae	Phyllophilopsis sp. 5	0	0	0	0	2
Tachinidae	Exoristinae	Sphaerina sp. 1	0	0	0	0	1
Tachinidae	Exoristinae	cf. Sphaerina sp. 1	0	1	0	0	0
Tachinidae	Exoristinae	aff. Sphaerina sp. 1	0	1	0	0	0
Tachinidae	Exoristinae	Thelyoxynops sp. 1	0	0	0	0	2
Tachinidae	Exoristinae	aff. Thelyoxynops sp. 1	0	0	1	0	0
Tachinidae	Exoristinae	Trigonospila sp. 1	0	0	1	0	10
Tachinidae	Exoristinae	cf. Trigonospila sp. 1	0	1	0	0	1
Tachinidae	Exoristinae	Vibrissina sp. 1	0	0	0	0	1
Tachinidae	Exoristinae	Vibrissina sp. 2	0	0	0	0	2
Tachinidae	Exoristinae	Zaira sp. 1	0	1	0	0	0
Tachinidae	Exoristinae	Carcelia sp. 1	0	2	0	0	0
Tachinidae	Exoristinae	Carcelia sp. 2	1	0	0	0	0
Tachinidae	Exoristinae	Drino sp. 1	0	0	2	0	2
Tachinidae	Exoristinae	Lespesia sp. 1	0	3	0	0	0
Tachinidae	Exoristinae	Lespesia sp. 2	0	0	2	0	0
Tachinidae	Exoristinae	Lespesia sp. 3	0	0	0	2	5
Tachinidae	Exoristinae	Siphosturmia sp. 1	0	2	0	0	0
Tachinidae	Exoristinae	Argyrophylax sp. 1	0	0	1	0	0
Tachinidae	Exoristinae	Atacta sp. 1	0	0	0	0	1
Tachinidae	Exoristinae	Atactosturmia sp. 1	0	0	0	5	8
Tachinidae	Exoristinae	Belvosia sp. 1	0	0	0	0	6
Tachinidae	Exoristinae	Chrysoexorista sp. 1	1	0	1	0	0
Tachinidae	Exoristinae	Distichona sp. 1	0	0	0	0	10
Tachinidae	Exoristinae	Hyphantrophaga sp. 1	0	5	1	0	0

Tachinidae	Exoristinae	Hyphantrophaga sp. 2	0	1	0	0	0
Tachinidae	Exoristinae	Houghia sp. 1	1	1	0	0	0
Tachinidae	Exoristinae	Patelloa sp. 1	0	0	0	7	2
Tachinidae	Exoristinae	Patelloa sp. 2	0	0	0	0	1
Tachinidae	Exoristinae	Pseudochaeta sp. 1	0	1	0	0	0
Tachinidae	Exoristinae	Masiphya sp. 1	0	0	1	0	0
Tachinidae	Exoristinae	Triodontopyga sp. 1	1	0	0	0	0
Tachinidae	Exoristinae	Winthemia sp. 1	0	1	3	2	2
Tachinidae	Exoristinae	Winthemia sp. 2	0	1	1	1	0
Tachinidae	Exoristinae	Austrophorocera sp. 1	0	1	0	0	4
Tachinidae	Exoristinae	cf. Chetogena sp. 1	0	0	0	0	2
Tachinidae	Exoristinae	Chetogena sp. 1	0	0	0	0	1
Tachinidae	Exoristinae	Chetogena sp. 2	0	0	0	0	1
Tachinidae	Exoristinae	cf. Pelecotheca sp. 1	0	0	0	0	4
Tachinidae	Exoristinae	cf. Pelecotheca sp. 2	0	0	4	0	0
Tachinidae	Exoristinae	*Cerotachina cf. albula*	0	0	0	0	2
Tachinidae	Exoristinae	*Cryptocladocera cf. prodigiosa*	0	2	0	0	0
Tachinidae	Exoristinae	Acemya sp. 2	0	0	0	0	1
Tachinidae	Tachininae	Phytomyptera sp. 1	0	0	0	0	36
Tachinidae	Tachininae	Phytomyptera sp. 2	0	0	0	2	6
Tachinidae	Tachininae	Phytomyptera sp. 3	0	0	1	6	4
Tachinidae	Tachininae	Phytomyptera sp. 4	0	0	0	1	1
Tachinidae	Tachininae	Phytomyptera sp. 5	0	0	1	54	23
Tachinidae	Tachininae	Phytomyptera sp. 6	0	0	2	3	28
Tachinidae	Tachininae	Phytomyptera sp. 7	0	1	1	1	5
Tachinidae	Tachininae	Phytomyptera sp. 8	1	0	34	6	5
Tachinidae	Tachininae	Icelia sp. 1	0	1	0	0	0
Tachinidae	Tachininae	Ginglymia sp. 1	0	0	4	0	0
Tachinidae	Tachininae	Leskia sp. 1	2	2	1	0	0
Tachinidae	Tachininae	Leskia sp. 2	0	1	1	0	0
Tachinidae	Tachininae	Leskia sp. 3	1	1	0	0	1
Tachinidae	Tachininae	Stomatodexia sp. 1	0	1	0	0	0
Tachinidae	Tachininae	*Uruleskia aff. aurescens*	0	8	0	1	0
Tachinidae	Tachininae	*Uruleskia infima*	0	8	1	0	0
Tachinidae	Tachininae	Leskiini gen. nov.	0	2	0	2	0
Tachinidae	Tachininae	Acronacantha sp. 1	0	0	1	0	0
Tachinidae	Tachininae	*Cholomyia acromion*	0	0	1	0	0
Tachinidae	Tachininae	Cholomyia sp. 1	0	0	0	0	1
Tachinidae	Tachininae	Unknown genus sp. 1	0	1	0	0	0
Tachinidae	Tachininae	Hypotachina sp. 1	0	1	0	0	0
Tachinidae	Tachininae	Xanthophyto sp. 1	0	2	1	1	9
Tachinidae	Tachininae	Xanthophyto sp. 2	0	1	0	0	0
Tachinidae	Tachininae	Xanthophyto sp. 3	0	0	0	1	0
Tachinidae	Tachininae	Xanthophyto sp. 4	0	1	1	2	3
Tachinidae	Tachininae	Xanthophyto sp. 5	0	2	0	0	0
Tachinidae	Tachininae	Ceromya sp. 1	0	1	1	0	0
Tachinidae	Tachininae	Ceromya sp. 2	0	6	1	0	1
Tachinidae	Tachininae	Ceromya sp. 3	0	7	2	3	0
Tachinidae	Tachininae	Ceromya sp. 4	0	13	1	0	0
Tachinidae	Tachininae	Ceromya sp. 5	0	0	3	0	0
Tachinidae	Tachininae	Pseudosiphona sp. 1	3	3	0	0	0
Tachinidae	Tachininae	Pseudosiphona sp. 2	0	9	4	5	0
Tachinidae	Tachininae	Pseudosiphona sp. 3	1	14	7	1	0
Tachinidae	Tachininae	Pseudosiphona sp. 4	0	4	0	0	0
Tachinidae	Tachininae	Pseudosiphona sp. 5	3	0	0	0	0
Tachinidae	Tachininae	Siphona sp. 1	0	1	3	0	0
Tachinidae	Tachininae	Siphona sp. 2	2	0	0	0	0
Tachinidae	Tachininae	Archytas sp. 1	0	0	1	0	0
Tachinidae	Tachininae	Exopalpus sp. 1	0	3	2	0	0
Tachinidae	Tachininae	*Phosocephala metallica*	0	1	2	0	1
			3634	1536	866	1812	961
